# IFIT3 and IFIT2/3 promote IFIT1-mediated translation inhibition by enhancing binding to non-self RNA

**DOI:** 10.1093/nar/gky191

**Published:** 2018-03-15

**Authors:** Renata C Fleith, Harriet V Mears, Xin Yun Leong, Thomas J Sanford, Edward Emmott, Stephen C Graham, Daniel S Mansur, Trevor R Sweeney

**Affiliations:** 1 Division of Virology, Department of Pathology, University of Cambridge, Addenbrooke's Hospital, Hills Road, Cambridge, UK; 2 Laboratory of Immunobiology, Department of Microbiology, Immunology and Parasitology, Universidade Federal de Santa Catarina, Florianópolis, Brazil; 3 Universidade Federal de Minas Gerais, Belo Horizonte, Brazil

## Abstract

Interferon-induced proteins with tetratricopeptide repeats (IFITs) are highly expressed during the cell-intrinsic immune response to viral infection. IFIT1 inhibits translation by binding directly to the 5′ end of foreign RNAs, particularly those with non-self cap structures, precluding the recruitment of the cap-binding eukaryotic translation initiation factor 4F and ribosome recruitment. The presence of IFIT1 imposes a requirement on viruses that replicate in the cytoplasm to maintain mechanisms to avoid its restrictive effects. Interaction of different IFIT family members is well described, but little is known of the molecular basis of IFIT association or its impact on function. Here, we reconstituted different complexes of IFIT1, IFIT2 and IFIT3 *in vitro*, which enabled us to reveal critical aspects of IFIT complex assembly. IFIT1 and IFIT3 interact via a YxxxL motif present in the C-terminus of each protein. IFIT2 and IFIT3 homodimers dissociate to form a more stable heterodimer that also associates with IFIT1. We show for the first time that IFIT3 stabilizes IFIT1 protein expression, promotes IFIT1 binding to a cap0 Zika virus reporter mRNA and enhances IFIT1 translation inhibition. This work reveals molecular aspects of IFIT interaction and provides an important missing link between IFIT assembly and function.

## INTRODUCTION

The host innate immune response provides a first line defence against invading pathogens. Following infection, pathogen recognition receptors (PRRs) sense non-self, pathogen-associated molecular patterns (PAMPs) triggering signaling pathways that activate an immune response (reviewed in 1). Detection of viral signatures by PRRs, such as RIG I-like receptor sensing of double stranded RNA, induces production of Type I and Type III interferon (IFN). Through binding of cell surface IFN receptors and subsequent activation of the JAK-STAT pathway, IFN activates the transcription of hundreds of IFN-stimulated genes (ISGs) (2), many with known antiviral properties, priming neighbouring cells to restrict viral spread.

The interferon induced protein with tetratricopeptide repeats (IFIT) protein family, present in all vertebrates, include some of the most highly expressed ISGs. Different species have varying complements of IFITs, but most mammals possess IFIT1, IFIT1B, IFIT2, IFIT3 and IFIT5 (3). IFITs typically contain multiple IFN-stimulated response elements (ISREs) in their promoters (reviewed in 3) and are also induced directly by IFN-regulatory factor 3 (5), downstream of initial PRR activation. Recently, phylogenetic analysis revealed that rodents, including mice, have lost IFIT1 but Ifit1b has undergone duplication twice (Ifit1b2 and Ifit1b3) and Ifit3 once (Ifit3b) (6).

IFITs are composed of sequential tetratricopeptide repeat (TPR) motifs that form globular N- and C-terminal domains joined by a linker of variable flexibility (7–11). TPR motifs are frequently involved in protein-protein interactions and are commonly found in scaffolding proteins (12). The crystal structures of IFIT1 and IFIT5 revealed a positively charged pocket formed in the groove between the N and C domains that interacts with single-stranded RNA (8–10). IFIT1 RNA binding activity was first reported by Pichlmair *et al*. (13) who identified proteins in lysates from IFN stimulated cells that interacted with 5′-ppp RNAs. Subsequently, we and others demonstrated that IFIT1 tightly binds capped RNAs lacking methylation on the first cap-proximal nucleotide (cap0) with low nanomolar affinity (14–16). While similar positively charged tunnels in IFIT1 and IFIT5 interact with the phosphate backbone of bound RNAs (8,9,14), only IFIT1 possesses a large hydrophobic cavity at the rear of the tunnel that can accommodate the cap structure (9). These findings support a model whereby IFIT1 out-competes eukaryotic initiation factor (eIF) 4E/4F for binding to cap0-mRNAs, thereby inhibiting their translation. As host mRNAs are generally methylated on the first or first and second bases (cap1 and cap2, respectively), this selectivity offers a mechanism of recognising and blocking translation of non-self RNAs.

The expression of IFIT1 imposes a strict requirement on viruses that replicate in the cytoplasm and rely on cap-dependent translation to evolve and maintain mechanisms to avoid restriction by this ISG. For example, members of the *Flaviviridae* and *Coronaviridae* families that rely on cap-dependent translation to produce viral proteins from their single-stranded positive-sense RNA genomes, encode their own 2′-*O*-methyltransferases. Disruption of methyltransferase activity increases susceptibility of the flaviviruses West Nile virus, Japanese encephalitis virus and dengue virus (16–20) and the coronaviruses murine hepatitis virus and severe acute respiratory syndrome virus (21,22) to IFIT1 restriction. Interestingly, enzymatically 2′-*O*-methylated, capped mRNAs from parainfluenza virus 5 display differential translational sensitivity to IFIT1 *in vitro*, while wild type middle east respiratory syndrome coronavirus (MERS) replication was enhanced upon IFIT1 depletion suggesting that factors other than 5′ end methylation can influence IFIT1 recognition (23,24). By contrast, alphaviruses, such as the emerging human pathogen chikungunya virus, also rely on cap-dependent translation but lack a virally encoded 2′-*O*-methyltranferase, thus possessing viral mRNAs with a cap0 structure at the 5′ end (25). Recent evidence suggests that stable secondary structure at the 5′ end of alphaviral genomes protects the viral RNAs from IFIT1 restriction (26,27). IFIT1 may also affect translation through interaction with eIF3 (28), while its direct binding to the viral E1 protein restricts human papilloma virus replication (29).

An intriguing feature of IFITs is their propensity to homo and heterooligomerize. Using pull-down experiments of differentially tagged IFITs, Stawowczyk *et al*. (30) demonstrated that IFIT2 could interact both with itself and with IFIT1 and IFIT3 in HeLa cells. In the same study, an IFIT1, IFIT2 and IFIT3 containing complex in HeLa cytoplasmic lysates was also reported to migrate between 150–200 kDa when analyzed by glycerol gradient sedimentation. Deletion analysis identified the first four TPRs of IFIT2 as being important for interaction with IFIT3 while the TPR(s) of IFIT2 that promote interaction with IFIT1 could not be elucidated (30). IFIT1 was later reported to interact with IFIT2 or IFIT3 by size exclusion chromatography (SEC) (13). The crystal structure of IFIT2 revealed it forms a stable, domain-swapped dimer, with TPRs of the N-terminal domain exchanged between each monomer (7). Using native gel electrophoresis, we previously demonstrated IFIT1 and IFIT3 could homooligomerize (14), while a recently-reported crystal structure of IFIT1 identified a motif in the C terminus responsible for IFIT1 dimerisation (BioRxiv: https://doi.org/10.1101/152850). However, despite considerable evidence for IFIT oligomerisation, little is known about how different IFITs interact and what impact this interaction has on function.

Here, we have reconstituted different IFIT complexes from individually purified proteins. We describe an assembly pathway for the IFIT heterocomplex identifying a critical motif for IFIT1 and IFIT3 interaction. We also demonstrate that IFIT1 expression is enhanced by the presence of other IFIT proteins and that interaction with IFIT3 or a heterocomplex of IFIT2 and IFIT3 enhances the cap0 RNA binding and translation inhibition activity of IFIT1. Our results provide a critical missing link between IFIT oligomerization and function presenting a mechanistic framework for understanding the role of IFITs in the host immune response.

## MATERIALS AND METHODS

### Plasmids

For mammalian cell expression, sequences for human IFIT1 (BC007091.1), IFIT2 (NM_001547.4), IFIT3 (NM_001549.5) and IFIT5 (BC025786.1) were PCR amplified to include a 5′ Kozak sequence, 3′ FLAG tag and 5′ BamHI and 3′ XhoI site to facilitate cloning into pCDNA3.1. The plasmid for expression of IFIT1 in *Escherichia coli* was previously described (10) and was used as a template for site directed mutagenesis to generate the mutant IFIT1 expression vectors. Sequences for IFIT2 and IFIT3 were PCR amplified to contain 5′ NdeI and 3′ XhoI restriction sites for cloning into pET28b (Novagen) producing a full-length protein with a thrombin cleavable N-terminal 6-His tag. Mutant IFIT3 expression vectors were generated by site directed mutagenesis using pCDNA3.1-IFIT3-FLAG or pET28b-IFIT3-His as templates. For reporter RNA transcription, the firefly luciferase reporter gene (Fluc) was PCR amplified using primers containing the 5′ UTR and 3′ UTR sequences of human β-globin (NM_000518.4), including a 5′ T7 promotor and EcoRI and PstI sites to facilitate cloning into pUC57. pUC57-ZIKV-Fluc was previously described (31). The Nano luciferase reporter gene (Nanoluc) flanked by the 5′ UTR and 3′ UTR sequences of the PE243 strain of Zika virus (ZV), including a 5′ T7 promoter sequence, was synthesized by Integrated DNA Technologies. EcoRI and HindIII sites were included to allow cloning into pUC57.

### Protein expression and purification

Recombinant IFITs were expressed in Rosetta 2 (DE3) pLysS *Escherichia coli* (Novagen). Cells were grown to an OD_600_ of approximately 1 in 2× TY media at 37°C. Expression was induced by adding 1 mM isopropyl β-d-1-thiogalactopyranoside. The induced culture was incubated at 22°C for 16 h. Cells were harvested and lysed in a buffer containing 20 mM Tris pH 7.5, 400 mM KCl, 5% glycerol, 1 mM DTT and 0.5 mM phenylmethylsulfonyl fluoride and 0.5 mg/ml lysozyme (from hen egg). IFITs were isolated by affinity chromatography on Ni-NTA Agarose beads (Qiagen). IFIT1 and IFIT1 mutants were additionally purified by FPLC on MonoQ (Q buffer: 20 mM Tris pH 7.5, 5% glycerol, 1 mM DTT and 100–500 mM KCl), followed by MonoS 5/50 GL (S buffer: 30 mM HEPES pH 7.5, 5% glycerol, 1 mM DTT and 100–500 mM KCl). IFIT2 and IFIT3 were treated with thrombin (from bovine plasma). IFIT2 and IFIT3 were further purified on MonoQ 5/50 (Q buffer), followed by size exclusion chromatography (SEC) on Superdex 200 increase 10/300 GL or HiLoad 16/600 Superdex 200 pg columns (SEC buffer: 20 mM Tris pH 7.5, 150 mM KCl and 1 mM DTT). All FPLC columns are from GE Healthcare and all buffer reagents and enzymes were from Sigma.

### *In vitro* IFIT complex assembly

All complexes were assembled in SEC buffer.

**IFIT1:IFIT2** and **IFIT1:IFIT3**—1 mg/ml of each protein was mixed and incubated for 1 h at 4 or 30°C. **IFIT2:IFIT3**—0.18 mg/ml of each protein was mixed and incubated for 1 h at 37°C and concentrated to 2 mg/ml. **IFIT1:IFIT2:IFIT3 trimer**—0.4 mg/ml of purified IFIT2:IFIT3 complex and 0.2 mg/ml of IFIT1 were incubated for 1 h at 30°C. The complex was concentrated to 3 mg/ml. **IFIT1:IFIT2:IFIT3 tetramer**—0.4 mg/ml of purified IFIT2:IFIT3 complex and 0.4 mg/ml of IFIT1 were incubated for 1 h at 30°C. The complex was concentrated to 4 mg/ml. Complexes were concentrated using Amicon Ultra 0.5 ml 10 kDa molecular weight cut off filters (Millipore).

### SEC analysis of mutant IFIT1 and IFIT3 complexes

Wild type or mutant IFIT1 and IFIT3 were combined as described in *IFIT complex assembly*. 150 μl of each assembly reaction was injected on to a Superdex 200 Increase 10/300 GL column, at 0.3 ml/min flow rate and UV280 readings were monitored. Peak fractions were analyzed by SDS-PAGE and Coomassie staining.

### SEC-multi-angle light scatter (SEC-MALS)

Proteins/complexes were injected (100 μl at the concentration described in *IFIT complex assembly*) onto an analytical Superdex 200 Increase 10/300 gel filtration column. MALS analysis was performed at room temperature, by inline measurement of static light scattering (DAWN 8+, Wyatt Technology), differential refractive index (Optilab T-rEX, Wyatt Technology), and 280 nm absorbance (Agilent 1260 UV, Agilent Technologies) following SEC at a flow rate of 0.4 ml/min. Molecular masses were calculated using the ASTRA6 software package (Wyatt Technology).

### Differential scanning fluorimetry

Differential scanning fluorimetry experiments to determine the thermal stability of different IFIT complexes was performed using a Viia7 Real-Time PCR system (Applied Biosystems). In an optical 96-Well Reaction Plate (Applied Biosystems 4366932), 1:500 Protein Thermal Shift dye (Life Technologies, 4461146) was mixed with 0.1 mg/ml protein in a final buffer composition of 20 mM HEPES pH 7.5, 150 mM KOAc, 2.5 mM MgOAc, 5% glycerol and 1 mM DTT in a final volume of 20 μl. Emission from quadruplicate samples was measured at 623 nm while ramping from 25 to 95°C stepwise at a rate of 1°C per 20 s. To determine *T*_m_, data were analyzed by non-linear regression using the Boltzmann equation *y* = LL + (UL – LL)/(1 + exp(*T*_m_ – *x*)/*a*) where LL and UL are the minimum and maximum fluorescence intensities respectively (32).

### *In vitro* transcription

pUC57-globin-Fluc was linearized with FspI. pUC57-ZIKV-Fluc and pUC57-ZIKV-Nanoluc were linearized with HindIII. RNA was transcribed using recombinant T7 polymerase at a final concentration of 50 ng/μl in transcription buffer (40 mM HEPES pH 7.5, 32 mM MgOAc, 40 mM DTT, 2 mM Spermidine, 10 mM NTPs, 0.2 U/μl RNaseOUT (Invitrogen)) for 2–4 h at 37°C. RNA was purified by DNaseI treatment, acidic phenol extraction and ethanol precipitation. Residual nucleotides were removed using Illustra MicroSpin G-50 columns (GE Healthcare). RNA was capped using the ScriptCap and ScriptCap 2′-*O*-methyltransferase system (CellScript).

### *In vitro* translation

IFIT proteins or complexes were diluted in bovine serum albumin (BSA) diluent buffer (0.5 mg/ml BSA, 20 mM Tris pH 7.5, 160 mM KCl, 5% glycerol, 2 mM DTT, 1 U/μl RNaseOUT), and incubated with 4 nM reporter RNA for 15 min at 37°C to allow RNA binding. *In vitro* translation was performed using the Flexi Rabbit Reticulocyte Lysate (RRL) System (Promega) for 90 min at 30°C. Reactions were terminated by incubation on ice, followed by addition of 50 volumes of passive lysis buffer (Promega) before luciferase signal was measured by GloMax (Promega). Luciferase values were normalized to the diluent buffer-only control for each experiment.

### Western blotting

Proteins were resolved by 12.5% SDS-PAGE and transferred to 0.45 μm nitrocellulose membrane. Antibodies used were anti-FLAG M2-Peroxidase (A8592, Sigma), anti-FLAG M2 (F3165, Sigma), anti-IFIT1 (PA3-848, ThermoFisher), anti-IFIT2 (12604-1-AP, Proteintech), anti-IFIT3 (PA5-22230, ThermoFisher), anti-GAPDH (AM4300, ThermoFisher) and anti-penta-His (34660, Qiagen). For pull-down experiments FLAG-tagged proteins were detected by chemiluminescence using Westar Supernova substrate (Cyanagen) and visualized on Super RX-N film (Fujifilm). For all other western blot experiments an Odyssey CLx Imaging System (Li-Cor) was used. To normalize recombinant IFIT proteins and complexes, membranes were probed with anti-penta-His and quantified using ImageJ.

### Analysis of the IFIT1-mRNA interaction by inhibition of primer extension (toeprinting)

IFIT1/mRNA interaction was performed essentially as described previously (14) with minor modifications. Cap0-ZV or cap0-β-globin reporter mRNAs (1 nM) were incubated with IFIT1 or IFIT1 containing complexes (at concentrations indicated in figures) for 10 min at 37°C in 20 μl reactions containing 20 mM Tris pH 7.5, 100 mM KCl, 2.5 mM MgCl_2_, 1 mM ATP, 0.2 mM GTP, 1 mM DTT, 0.25 mM spermidine and 0.5 mg/ml BSA. IFIT1/mRNA interaction was monitored by inhibition of primer extension using avian myeloblastosis virus reverse transcriptase (2.5 U) (Promega) and a ^32^P-labeled primer in the presence of 4 mM MgCl_2_ and 0.5 mM dNTPs. Full length and truncated cDNA products were separated in a denaturing 6% acrylamide gel and detected by autoradiography using an FLA7000 Typhoon Scanner (GE). Analysis was performed using Image-Quant TL.

### HEK293T transfection

HEK293T cells (1 × 10^6^) were transfected with wild type or mutant IFIT1, IFIT2 and/or IFIT3 FLAG-tagged expression constructs as indicated in the text using Lipofectamine 2000 (ThermoFisher) using the manufacturers standard protocol. After 24 h cells were harvested, and protein expression analyzed by western blotting.

### RNA transfection

HEK293T cells (1 × 10^6^) were transfected with wild type IFIT1 and wild type or mutant IFIT3 expression plasmids as indicated in the text using Lipofectamine 2000 (ThermoFisher). After 24 h, cells were trypsinized and 1.5 × 10^5^ cells per well were plated into a 48-well plate, in duplicate. After 4 h, cells were washed into Opti-MEM medium (Thermofisher) and transfected with 100 fmol each cap0-ZIKV-Fluc and cap1-ZIKV-Nanoluc RNA using Lipofectamine 2000, or untreated (mock), for 6 h. Cells were harvested in passive lysis buffer. Fluc signal was detected as described above. Nanoluc signal was detected using the Nano-Glo luciferase assay system (Promega). Plasmid transfection was performed in triplicate and RNA transfections in duplicate. Luciferase values are expressed as a ratio of Nanoluc (cap1) over Fluc (cap0), normalized to the empty vector control.

### Stable isotope labeling with amino acids in cell culture and immunoprecipitation

HEK293T cells were cultured in Arg/Lys-free DMEM, supplemented with light (R0K0), medium (R6K4) or heavy (R10K8) amino acids, as described (33). 1 × 10^7^ cells were transfected with 10 μg plasmid DNA using Lipofectamine 2000 (ThermoFisher). After 24 h, media was replaced to contain 1000 U/ml human interferon α-2a (Roferon-A, Roche) for a further 16 h. Cells were harvested in lysis buffer (50 mM Tris pH 7.5, 150 mM NaCl, 1 mM EDTA, 1% Triton X100) containing 1:200 Protease Inhibitor Cocktail Set III (Merck) and 1:200 Benzonase nuclease (Sigma-Aldrich). Lysates were normalized to 3 mg/ml of protein before incubation with anti-FLAG-M2 affinity gel (Sigma) at 4°C for 18 h. Beads were washed three times in Tris-buffered saline, then resuspended in 2× SDS-sample buffer (Invitrogen) and boiled for 5 min to elute bound proteins.

### LC–MS/MS and sample preparation

Following immunoprecipitation, the combined samples were subjected to SDS-PAGE electrophoresis on a precast gel, and extracted as a single band for in-gel trypsinisation. The resulting peptides were fractionated using an Ultimate 3000 nanoHPLC system in line with an Orbitrap Fusion Tribrid mass spectrometer (Thermo Scientific). In brief, peptides in 1% (vol/vol) formic acid were injected onto an Acclaim PepMap C18 nano-trap column (Thermo Scientific). After washing with 0.5% (vol/vol) acetonitrile 0.1% (vol/vol) formic acid, peptides were resolved on a 250 mm × 75 μm Acclaim PepMap C18 reverse phase analytical column (Thermo Scientific) over a 150 min organic gradient, using seven gradient segments (1–6% solvent B over 1 min, 6–15% B over 58 min, 15–32% B over 58 min, 32–40% B over 5 min, 40–90% B over 1 min, held at 90% B for 6 min and then reduced to 1% B over 1 min) with a flow rate of 300 nl min^−1^. Solvent A was 0.1% formic acid and Solvent B was aqueous 80% acetonitrile in 0.1% formic acid. Peptides were ionized by nano-electrospray ionization at 2.0 kV using a stainless steel emitter with an internal diameter of 30 μm (Thermo Scientific) and a capillary temperature of 275°C.

All spectra were acquired using an Orbitrap Fusion Tribrid mass spectrometer controlled by Xcalibur 2.1 software (Thermo Scientific) and operated in data-dependent acquisition mode. FTMS1 spectra were collected at a resolution of 120 000 over a scan range (*m/z*) of 350–1550, with an automatic gain control (AGC) target of 300 000 and a max injection time of 100 ms. Precursors were filtered using an Intensity Range of 1E4 to 1E20 and according to charge state (to include charge states 2–6) and with monoisotopic precursor selection. Previously interrogated precursors were excluded using a dynamic window (40 s ±10 ppm). The MS2 precursors were isolated with a quadrupole mass filter set to a width of 1.4 *m/z*. ITMS2 spectra were collected with an AGC target of 20 000, max injection time of 40 ms and CID collision energy of 35%.

### Mass spectrometry data analysis

The raw data files were processed and quantified using MaxQuant v1.5.7.4 (34) and searched against the Uniprot Human database (70 550 entries, dated 19 September 2016) using the built-in Andromeda search engine. Peptide precursor mass tolerance was set a 4.5 ppm, and MS/MS tolerance was set at 0.5 Da. Search criteria included carbaminomethylation of Cys as a fixed modification. Oxidation of Met and N-terminal acetylation were selected as variable modifications. Quantification was based on Light (Arg 0, Lys 0), Medium (Arg 6, Lys 4), and Heavy (Arg 10, Lys 8) SILAC labels. Searches were performed with tryptic digestion, a minimum peptide length of seven amino acids, and a maximum of two missed cleavages were allowed. The reverse database search option was enabled and the maximum false discovery rate for both peptide and protein identifications was set to 0.01. Quantitation was performed using a mass precision of 2 ppm. The full MaxQuant output is provided as part of PRIDE submission PXD007584 permitting viewing of annotated spectra in MaxQuant v1.5.7.4. Downstream analysis was accomplished in the Perseus software (35). Contaminants and reverse database hits were removed, and protein ratios were log_2_-transformed. Proteins were considered to represent putative interaction partners if they showed a significant (*t*-test, *P* < 0.05) increase in their abundance compared with the control pulldown and had to have been identified in at least two of the three replicates. Full proteomics data sets are available on ProteomeXchange via the PRIDE repository with the identifier PXD007584.

### Protein structure modeling and analysis

Protein structure analysis and generation of protein structure images was performed using PyMOL (The PyMOL Molecular Graphics System, Version 2.0 Schrödinger, LLC). The IFIT3 model was generated by submitting the IFIT3 amino acid sequence to the Swiss Model server. The crystal structure of the IFIT2 domain-swapped homodimer (PDB:4G1T) was used as a template for model generation. The electrostatic surface potential of IFIT2 and the IFIT3 model were analyzed using PDB2PQR and APBS software (36–38).

## RESULTS

### IFIT1 co-precipitates IFIT2 and IFIT3 independently of RNA association

**Figure 1.**
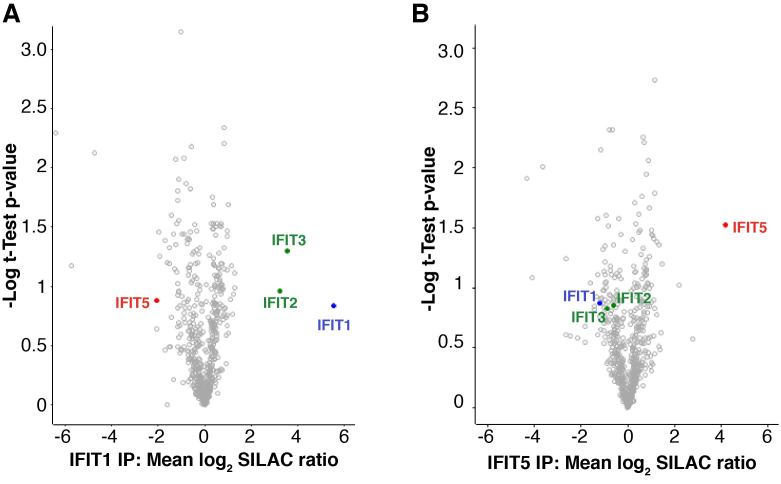
Interaction between IFIT1, IFIT2 and IFIT3 occurs independently of bound RNA. Volcano plots of SILAC proteomics data showing statistical analysis of proteins that immuno-precipitated with (**A**) FLAG-tagged IFIT1 or (**B**) FLAG-tagged IFIT5 following nuclease treatment of IFNα-stimulated cell lysates. Data represent the analysis of three biological replicate experiments, and the mean log_2_-fold change and t-test *P*-values plotted represent the combined data from these three replicates. Data points corresponding to IFIT family members are colored and labeled.

IFIT1 was originally identified as an RNA binding protein after being precipitated by 5′-ppp RNA from lysates of IFN stimulated HEK293 cells. IFIT2 and IFIT3, as well as several well-characterized RNA binding proteins, co-precipitated with IFIT1 (13). We used SILAC proteomics to examine if IFIT1 could interact directly with IFIT2 and IFIT3 in nuclease treated lysates from IFN-stimulated cells. HEK293T cells were passaged in differentially isotopically labeled media and transfected with either a plasmid expressing FLAG-tagged IFIT1, FLAG-tagged IFIT5 (previously reported not to interact with other IFITs (13,30)), or an empty vector control. Twenty four hours after transfection the cells were treated with IFN-α and incubated for a further 16 h. Preparation of nuclease treated cell lysates and pull-down experiments are described in Materials and Methods. Consistent with previous reports (39), IFIT1 was poorly overexpressed while IFIT5 was strongly expressed (Supplementary Figure S1). However, as shown in Figure 1A, IFIT2 and IFIT3 co-precipitated with FLAG-tagged IFIT1, while FLAG-tagged IFIT5 did not precipitate other IFIT family members (Figure 1B). The full SILAC data set is available on the PRIDE server. These results independently confirm the interaction of IFIT1, 2 and 3 in IFN-stimulated cell lysates and further demonstrate that this interaction is maintained after nuclease treatment. Both IFIT2 and IFIT3 were enriched to a similar extent in the IFIT1 pull downs (Figure 1A).

### IFIT1:IFIT2:IFIT3 hetero-complexes can be reconstituted from bacterially expressed proteins

To investigate human IFIT family oligomerisation and examine the influence of interaction with IFIT2 and IFIT3 on IFIT1 mRNA cap0 binding activity we reconstituted the IFIT1:IFIT2:IFIT3 hetero-complex *in vitro*. To this end, His-tagged IFIT1, IFIT2 and IFIT3 were individually expressed in bacteria and purified as described in Materials and Methods. The His-tag was removed from IFIT2 and IFIT3 but retained on IFIT1 for later detection. Each protein was subjected to size exclusion chromatography with multi angle light scattering (SEC-MALS) to analyze IFIT assembly. SEC-MALS reveals the molecular mass of species eluting at different volumes from a size exclusion column providing information about the oligomeric state of these species. Varying concentrations of IFIT were analyzed by SEC-MALS and, consistent with a recent report (BioRxiv: https://doi.org/10.1101/152850), IFIT1 oligomerized in a concentration-dependent manner (Supplementary Figure S2A). In contrast, IFIT2 eluted as two species corresponding to a stable dimer or tetramer (Supplementary Figure S2B). Surprisingly, the IFIT2 dimer had a similar elution volume to the lowest concentration of IFIT1 examined. This demonstrates the importance of using the SEC-MALS technique which directly determines the mass of particles in solution from their Rayleigh scattering, instead of relying on the elution volumes of molecular weight standards during SEC to infer oligomeric state. When analyzed alone, IFIT3 also eluted as a mostly dimeric species, with a smaller peak corresponding to a monomer (Supplementary Figure S2C).

**Figure 2.**
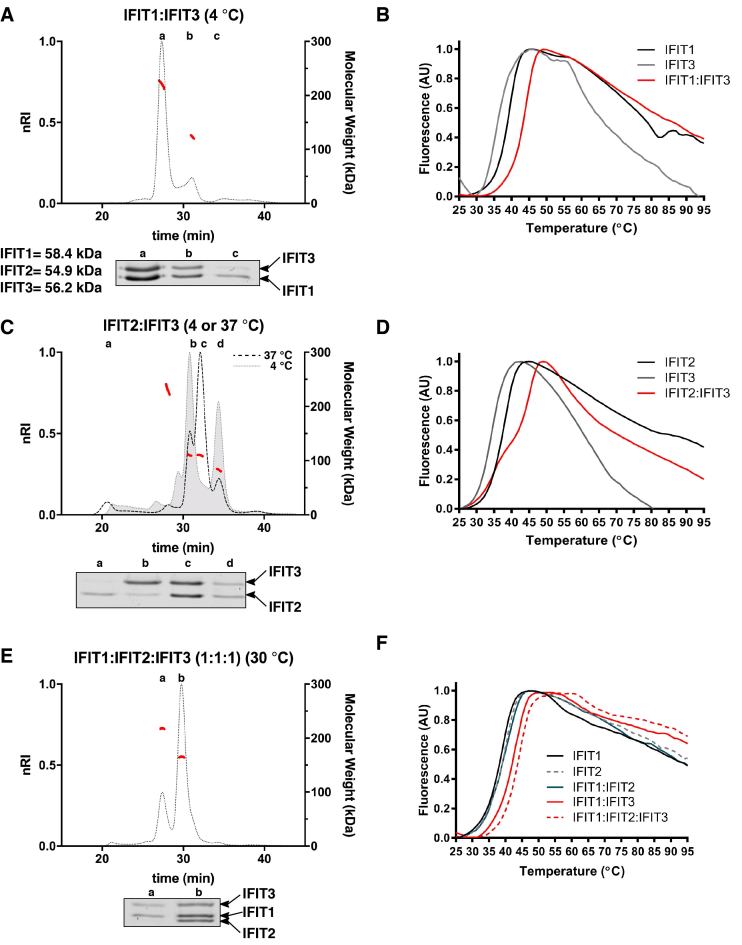
*In vitro* reconstitution of IFIT oligomeric assemblies. (**A, C** and **E**) Indicated IFIT complexes were assembled and analyzed by SEC-MALS as described in the Materials and Methods. Complexes were formed at the indicated temperatures. Normalized differential refractive index (nRI) is shown as dotted or broken lines on the left *y-axis*. Calculated molecular masses (kDa) of eluting species are shown as solid, red lines on the right *y-axis*. Gel insets below each trace show SDS-PAGE analysis of each run. Protein gel lanes and corresponding peaks are indicated by lower case letters. The position of IFIT1, IFIT2 and IFIT3 on the protein gels is indicated. (**B, D** and **F**) Differential scanning fluorimetry analysis of the indicated complexes was performed as described in the Materials and Methods.

We next examined the oligomeric status of complexes containing mixtures of the individually purified IFIT proteins. IFIT1 and IFIT3 formed a stable complex that eluted with a molecular mass of 221 kDa (Figure 2A, peak a). Analysis of the protein peaks by SDS-PAGE shows that IFIT1 and IFIT3 are equimolar, indicating that this complex represents stable IFIT1:IFIT3 tetramers. A later eluting species likely corresponding to IFIT1:IFIT3 dimers (123 kDa) is also evident (Figure 2A, peak b). To determine the relative stabilities of IFIT1 alone or complexed with IFIT3 we examined IFIT1 and the IFIT1:IFIT3 heterocomplex using differential scanning fluorimetry. Using this approach, protein unfolding is monitored by measuring the signal from a dye that fluoresces in hydrophobic environments at increasing temperature. More stable proteins unfold at higher temperatures than less stable proteins. Unfolding exposes hydrophobic regions of the protein making them more accessible to the dye. As seen in Figure 2B the IFIT1:IFIT3 complex melts at a higher temperature than either IFIT1 or IFIT3 in isolation indicating that the complex is indeed more stable than either protein alone. The IFIT1:IFIT2 complex was less defined than IFIT1:IFIT3 and eluted as multiple species ranging from 148 to >234 kDa as measured by SEC-MALS (Supplementary Figure S2D). Interestingly this interaction was only observed when the proteins were incubated at 30°C prior to SEC-MALS analysis. When combined at 4°C prior to SEC analysis only a very weak interaction between IFIT1 and IFIT2 was observed (Supplementary Figure S2E).

Because IFIT2 and IFIT3 were enriched to a similar degree in our IFIT1 pull down SILAC experiments we hypothesized that IFIT1 could interact with a heterodimer of IFIT2 and IFIT3. We therefore examined if IFIT2 and IFIT3 formed a stable complex. Equal amounts of IFIT2 and IFIT3 were mixed and incubated at 4°C for 1 h. When analyzed by SEC-MALS two peaks corresponding to IFIT2 and IFIT3 homodimers were clearly separated (Figure 2C, grey dotted line). In contrast, when IFIT2 and IFIT3 were instead incubated at 37°C for 1 hour an IFIT2:IFIT3 heterodimer was formed (Figure 2C, black dashed line). The molecular weight of the eluting species (110 kDa) and analysis by SDS-PAGE (gel inset) are consistent with this complex representing an IFIT2:IFIT3 heterodimer. As evident in Supplementary Figure S2F, when peak fractions were reanalyzed by SEC-MALS there was no observed dissociation of the complex into constituent components, confirming that the IFIT2:IFIT3 complex is stable. To determine the relative stabilities of the different dimeric complexes, we examined IFIT2, IFIT3 and the IFIT2:IFIT3 heterodimer using differential scanning fluorimetry. Figure 2D shows the thermal melt curves for IFIT2 and IFIT3 homodimers and the IFIT2:IFIT3 heterodimer. Of the three sets of dimers IFIT3 was the least stable as it displays the lowest melting temperature while IFIT2:IFIT3 was more stable than either homodimer.

Having successfully formed a stable IFIT2:IFIT3 complex, we attempted to reconstitute the full IFIT1:IFIT2:IFIT3 heterotrimeric complex. An equimolar ratio of IFIT1 was incubated with the purified (preformed) IFIT2:IFIT3 heterodimeric complex. Most of the IFIT1 was incorporated into a larger complex that eluted with a molecular mass of 165 kDa corresponding to a trimer (Figure 2E). Analysis of this complex by SDS-PAGE (gel inset) reveals that it contains equimolar amounts of IFIT1, IFIT2 and IFIT3. Finally, we incubated the IFIT2:IFIT3 heterodimer with a two-fold molar excess of IFIT1 at 30°C for 1 hour. Analysis by SEC-MALS reveals a clear shift of the heterotrimeric complex to a heterotetrameric complex with a molecular mass of 236 kDa (Supplementary Figure S2G). SDS-PAGE analysis of this complex reveals a molar excess of IFIT1 over IFIT2 and IFIT3 indicating that the heterotetrameric complex consists of two IFIT1 molecules to one molecule each of IFIT2 and 3 (compare gel insets in Figure 2E and Supplementary Figure S2G). The ability to form this complex *in vitro* from purified components demonstrates that the complex can assemble independently of IFN stimulation. The IFIT1:IFIT2:IFIT3 heterotetramer precipitated with time so the IFIT1:IFIT2:IFIT3 heterotrimer was used for further experiments. We examined the relative stability of each of our new complexes by differential scanning fluorimetry as shown in Figure 2F. The thermal melting curves for IFIT1, IFIT2 and the IFIT1:IFIT2 complex were nearly identical indicating that each of these are more stable than IFIT3 alone (compare Figure 2B and F) whereas IFIT1:IFIT3 and IFIT1:IFIT2:IFIT3 were the most stable complexes examined.

### IFIT3 and IFIT2:IFIT3 stimulate the cap0-RNA translation inhibition activity of IFIT1

**Figure 3.**
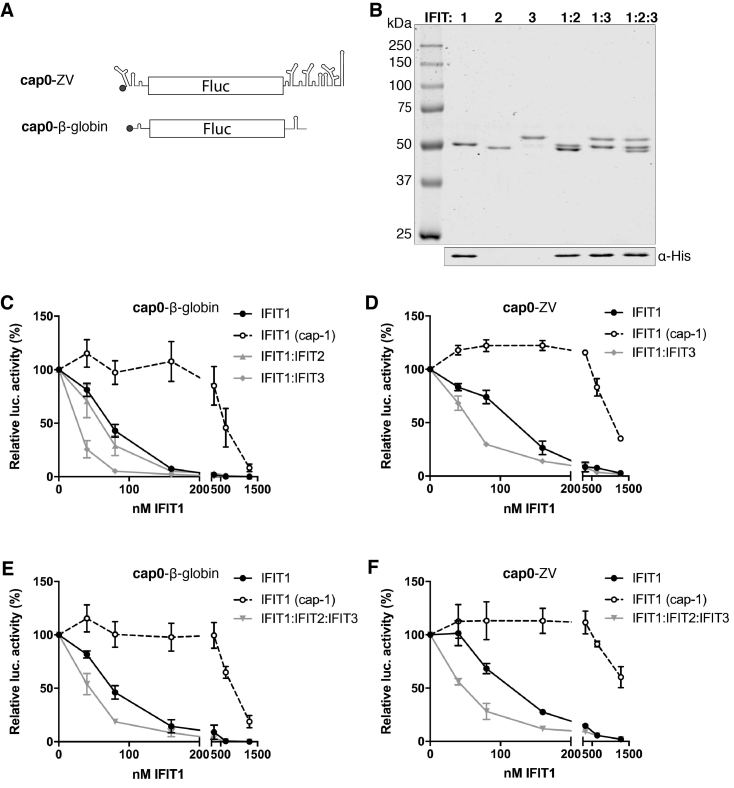
IFIT3 and IFIT2:IFIT3 enhance IFIT1 cap0 translation inhibition *in vitro*. (**A**) Schematic representation of the cap0-mRNAs used in *in vitro* translation assays in RRL. (**B**) IFIT1 containing complexes included in *in vitro* translation assays. *Upper panel*, Coomassie stained SDS-PAGE gel analysis of individually purified and complexed IFITs. *Lower panel*, analysis of the same samples as in the upper panel by western blotting against the His-tag. Note, the His-tag was removed from IFIT2 and IFIT3 but not IFIT1 during purification. (**C**–**F**) Luciferase activity from RRL incubated with cap0-β-globin Fluc RNA (C, E) or cap0-ZV Fluc RNA (D, F) in the presence of increasing concentrations of IFIT1 or IFIT1 containing complexes as indicated. In each panel (C-F) the effect of IFIT1 on the cap1 versions of the model RNAs is also shown. Data are normalized to the luciferase activity in the absence of IFITs and shown as the mean ± the standard error of three separate experiments. To calculate the IFIT complex concentration at which translation was inhibited by 50% data were fitted to [Inhibitor] versus normalized response curve (*Y* = 100/(1 + (*X*^HillSlope^)/(IC_50_^HillSlope^) using the least squares method in GraphPad Prism. IFIT concentrations at which reporter translation was 50% inhibited are reported as IC_50_ values in Table 1.

To investigate the impact of hetero-oligomerisation on the ability of IFIT1 to inhibit cap0-dependent translation we used a similar approach to that previously described by Young *et al*. (23). *In vitro* transcribed mRNAs comprising a firefly luciferase (Fluc) reporter flanked by either the ZV or human β-globin 5′ and 3′ untranslated regions (UTRs) were post-transcriptionally capped as described in the Materials and Methods. As described in the Introduction, flaviviruses lacking 2′-*O*-methylation are inhibited by IFN in an IFIT1-dependent manner. As such the cap0 version of this reporter is a representative mRNA that is inhibited by IFIT1. The β-globin reporter was examined as it has very weak secondary structure and a previous study has suggested that IFIT1 binding may be affected by both 5′ end methylation state and RNA secondary structure (9). Schematics of the two constructs are shown in Figure 3A. These mRNAs were incubated with different IFIT heterocomplexes before addition of rabbit reticulocyte lysate (RRL). Advantages of the RRL system for this analysis are that the impact of different IFIT complexes on translation can be monitored independently of other changes occurring due to IFN stimulation and that the effects of defined amounts of each complex can be examined. We also previously used translation factors purified from RRL for the initial demonstration that IFIT translation regulation was affected by the cap structure of the mRNA 5′ end (14). A well-known restriction of the RRL system is that it does not completely recapitulate the cap/polyA synergy of translation initiation (40). However, ZV, like other flaviviruses that utilize cap-dependent translation, do not possess 3′ polyA tails and do not require circularization for translation (41). Translation was quantified by measuring the luminescence from the Fluc reporter. The amount of IFIT1 added was equalized by western blotting against the His-tag on the purified IFIT1 in each complex. The upper panel of Figure 3B shows an SDS-PAGE analysis of the protein complexes used in these experiments while the lower panel shows an anti-His-tag western blot of the same complexes. The linearity of the western blot signal to protein concentration was confirmed as shown in Supplementary Figure S3.

IFIT1 inhibited translation of the cap0-β-globin Fluc reporter in a concentration-dependent manner (Figure 3C). Pre-incubation of IFIT1 and IFIT2 had no detectable impact on the inhibition of translation of the cap0-β-globin Fluc reporter and so was not examined further. In contrast, complexing with IFIT3 reproducibly decreased the concentration of IFIT1 required to cause 50% inhibition of the same reporter (Figure 3C). The IC_50_ values derived from the experiments shown in Figure 3 are displayed in Table 1. IFIT1 inhibited translation of the cap0-ZV reporter less than the β-globin reporter overall but complexing with IFIT3 again enhanced IFIT1’s translation inhibitory effect (Figure 3D). As can be seen in Figure 3E and F, stimulation of IFIT1 translation inhibition was reproducibly observed in the context of the IFIT1:IFIT2:IFIT3 complex. The average volume of HeLa cells was previously reported as 2.6 × 10^3^ μm^3^ (42), while 2.4 × 10^6^ copies of IFIT1 were estimated to be present after IFN stimulation of HeLa cells (13). This would mean IFIT1 is present at an effective concentration of ≥1.5 μM making the concentrations used in our assays physiologically relevant. Reporter mRNAs bearing cap1 were also examined (Figure 3C–F and Supplementary Figure S4) but showed much greater resistance to IFIT1 inhibition. The ability to observe this ∼two-fold decrease in the IC_50_ values for the inhibition of translation by IFIT1 in the presence of IFIT3 or IFIT2:IFIT3 in this system is significant, as RRL is very efficient in supporting cap-dependent translation as evidenced by the lack of cap/polyA synergy described above. Our results clearly implicate the structure present at the 5′ end of a mRNA, as well as the methylation state, as an important determinant of susceptibility to IFIT1 restriction and that interaction with IFIT3 or IFIT2:IFIT3 can also influence this.

**Table 1.** Comparison of translation inhibition and cap0 mRNA binding kinetics

Complex/RNA	IC_50_^†^ (nM IFIT1 in RRL)	*P*-value (IFIT1 vs complex)	RNA binding (*K*_1/2, app_, nM)	Hill number
IFIT1/cap0-β-globin	71 ± 5.9	-	40 ± 1.2	2.8 ± 0.2
IFIT1:IFIT2/cap0-β-globin	58 ± 5.3	0.2798	-	-
IFIT1:IFIT3/cap0-β-globin	24 ± 1.2	<0.0001	19 ± 0.8	2.5 ± 0.2
IFIT1:IFIT2:IFIT3/cap0-β-globin	45 ± 2.9	<0.0001	19 ± 0.7	2.0 ± 0.1
IFIT1/cap0-ZV	112 ± 7.6	-	69 ± 1.8	3.5 ± 0.3
IFIT1:IFIT3/cap0-ZV	54 ± 3.5	<0.0001	29 ± 1.1	4.1 ± 0.6
IFIT1:IFIT2:IFIT3/cap0-ZV	46 ± 2.6	<0.0001	58 ± 2.3	3.1 ± 0.3

**Figure 4.**
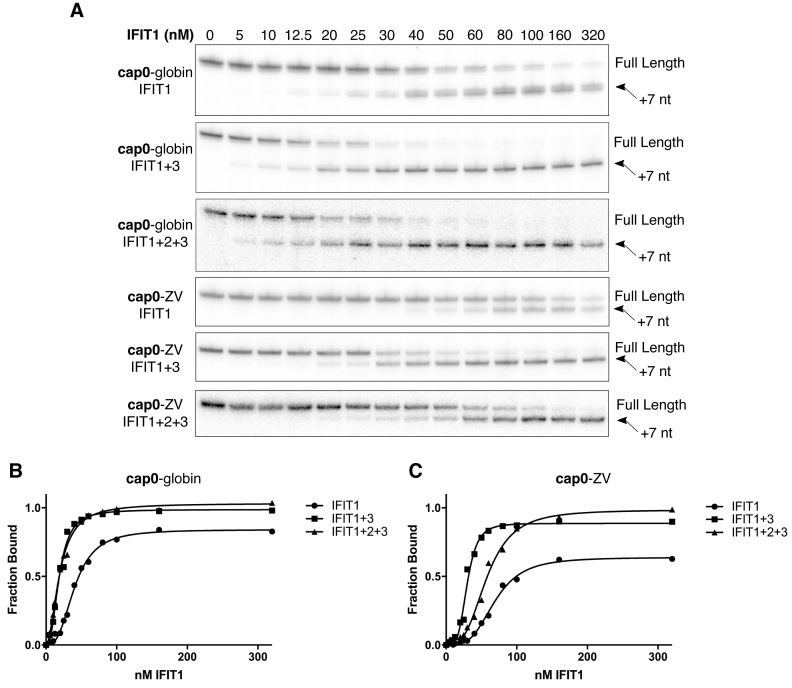
IFIT3 containing complexes stabilize IFIT1 binding to cap0 RNA. (**A**) Toeprinting analysis of the interaction of IFIT1 and IFIT1 containing complexes with cap0 RNA. The full-length and 7 nucleotide (nt) truncated cDNA product produced by IFIT1 binding are indicated. Protein complexes and RNAs are the same as those used in Figure 3. (**B, C**) Graphs represent fraction of RNA bound by IFIT1 and IFIT1 containing complexes at varying IFIT1 concentrations. Curves representative of three separate experiments were fitted using the nonlinear Hill equation, Fraction^[bound]^ = [IFIT1]^*h*^ • Fraction^[bound]^_max_/([IFIT1]*h* + *K^h^*_1/2,app_) from data where [IFIT1] was ≥ 10 • [mRNA]. *K*_1/2_,_app_ and Hill coefficients (*h*) are listed in Table 1.

Values are from data presented in Figures 3 and 4. Details of the analysis performed are included in the corresponding figure legends.

^†^IC_50_ values are the concentration of IFIT complexes that reduce the reporter translation by 50% ± standard error.

### IFIT3 and IFIT2:IFIT3 enhance cap0-RNA binding by IFIT1

We and others have previously demonstrated that IFIT1 binds preferentially to mRNA with a cap0 at the 5′ end (14,15). Using our purified complexes, we next examined if the interaction of IFIT1 with cap0 mRNA was altered when part of a larger IFIT1:IFIT3 or IFIT1:IFIT2:IFIT3 complex. We used a primer extension inhibition assay to monitor the IFIT1/cap0 mRNA interaction as previously described (14). An advantage of this technique over other methods to analyze protein–RNA interactions, such as electrophoretic mobility shift assays, is that the primer extension reaction is performed in equilibrium binding conditions. IFIT1 alone or as part of an IFIT heterocomplex was incubated with the *in vitro* transcribed and capped model β-globin and ZV mRNAs prior to the addition of a radiolabeled primer that binds within the Fluc mRNA sequence. A reverse transcription reaction was performed in which a full-length cDNA is produced in the absence of IFIT1, whereas a 7 nucleotide truncated cDNA, corresponding to the length of the IFIT1 RNA-binding surface, is produced in the presence of IFIT1 (14). The cDNA products are subsequently separated by denaturing PAGE and detected by autoradiography. IFIT protein complexes shown in Figure 3B were used in the binding reactions. Representative autoradiographs are shown in Figure 4A. Quantification of the cDNA products was performed as described in the Materials and Methods and the binding curves shown in Figure 4B and C show the fraction of RNA bound at varying IFIT concentrations.

As previously reported (14), IFIT1 binds cap0-β-globin mRNA with very high affinity. The binding constants for the experiments shown in Figure 4 are presented in Table 1. Complexing with IFIT3 or the IFIT2:IFIT3 heterodimer enhances binding of IFIT1 to this mRNA. IFIT1 heterocomplexing also has the effect of saturating the binding on the mRNA as evident in the autoradiograms. This effect was even more pronounced when the cap0-ZV reporter was analyzed. On this more structured model viral RNA IFIT1 binding only reached 60% saturation. When complexed with IFIT3, IFIT1 bound the ZV reporter with 2-fold higher affinity, similar to the effect observed for the cap0-β-globin mRNA. In contrast, the calculated *K*_1/2, app_ for the IFIT1:IFIT2:IFIT3 complex binding to the cap0-ZV reporter was only marginally lower than that for IFIT1 alone. However, again as is clear from the autoradiograms, addition of IFIT3 or the IFIT2:IFIT3 heterodimer led to saturation of RNA binding. It is not yet known how IFIT3 promotes IFIT1-cap0 binding, however, based on our *in vitro* RNA binding experiments we speculate that IFIT3 decreases the IFIT1 off rate, enhancing its ability to block eIF4F recruitment. This is potentially particularly important when IFIT1 is binding mRNA with highly structured 5′ ends as our translation inhibition results in Figure 3 suggest. As IFIT1:IFIT3 is predominantly a stable tetramer with two copies of each protein and since complexing with IFIT3 has a more noticeable effect on the apparent affinity of IFIT1 for cap0 mRNA, we conclude that IFIT3 and not IFIT2 in the IFIT2:IFIT3 complex is responsible for the observed effects. In all cases, and similar to our previous findings (14), the Hill coefficient was greater than 1, indicating a degree of cooperativity in IFIT1 cap0-mRNA binding.

### IFIT1 and IFIT3 interact through a C-terminal motif

**Figure 5.**
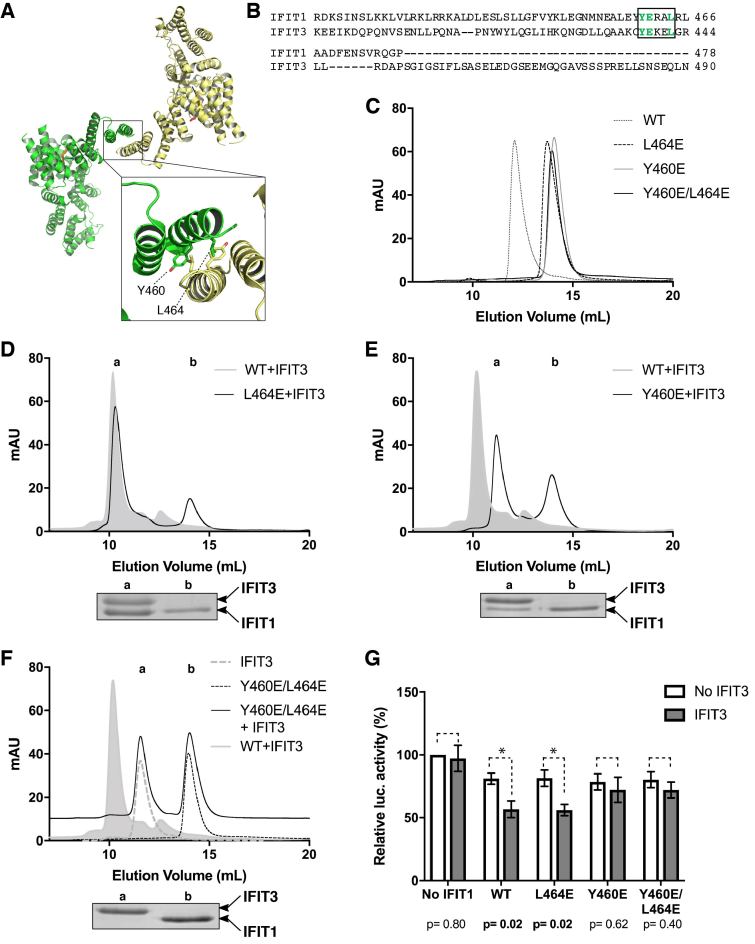
Mutational analysis reveals a key IFIT1:IFIT3 interaction motif. (**A**) Crystal structure of wild type IFIT1 (PDB: 5W5H). The two chains in the asymmetric unit are colored yellow and green with m7GpppAAAA bound in the cap-binding pocket highlighted in orange. The interface region between the two IFIT1 molecules with Y460 and L464 side chains is enlarged (only labeled for the chain colored green). (**B**) Sequence alignment of the C-terminal regions of IFIT1 and IFIT3 generated by Clustal Omega. The YxxxL motif is boxed. (**C**–**F**) UV_280_ absorbance traces of SEC analysis (SuperdexS200 Increase 10/300 column) of wild type (WT) and mutant IFIT1 alone or incubated with IFIT3. (D–F) Gel insets below each trace show SDS-PAGE analysis of each run. Protein gel lanes and corresponding peaks are indicated by lower case letters. The position of IFIT1 and IFIT3 on the protein gels is indicated. The Y460E/L464E+IFIT3 trace in F is adjusted by +10 milli absorbance units (mAU) for clarity. The elution profile of WT IFIT1+IFIT3 is shown in grey shadow for reference. (**G**) Luciferase activity from RRL incubated with cap0-β-globin Fluc RNA and WT or mutant IFIT1 with or without IFIT3 as indicated. Data are normalized to the luciferase activity in the absence of IFIT1 and shown as the mean ± the standard error of three separate experiments. Statistical analysis was performed using an unpaired, two-tailed Students T-test. *P* values are indicated, and ∗ denotes statistical significance.

Our data reveal that IFIT3 or the IFIT2:IFIT3 heterodimer can stimulate non-self mRNA binding and translation inhibition by IFIT1. We next sought to identify how IFIT1 and IFIT3 interact. Murine Ifit3, which does not precipitate with murine Ifit1b1 (15), has a large deletion at the C terminus (Supplementary Figure S5) when compared to human IFIT3. As a result of this deletion, mouse Ifit3 lacks a YxxxL structural motif present in both human IFIT3 and IFIT1 (Figure 5A and B) recently reported to promote IFIT1 concentration-dependent dimerisation (BioRxiv: https://doi.org/10.1101/152850). Our SEC-MALS and differential scanning fluorimetry analysis demonstrates that the IFIT1:IFIT3 interaction is more stable than the IFIT1:IFIT1 interaction (compare Figure 2A and Supplementary Figure S2A). We therefore hypothesized that the proposed IFIT1 dimerization motif is the site of interaction between IFIT1 and IFIT3.

Based on the IFIT1 dimer crystal structure (PDB: 5W5H) we generated three IFIT1 mutants, Y460E and L464E single mutants and a Y460E/L464E double mutant. All mutants expressed and purified similarly to the wild type protein and eluted as monomeric species during SEC (Figure 5C) consistent with disruption of IFIT1 homodimerization as previously reported (BioRxiv: https://doi.org/10.1101/152850). However, the L464E substitution had only a modest effect on the interaction of IFIT1 with IFIT3 (Figure 5D). The Y460E substitution destabilizes IFIT1:IFIT3 oligomerization to a greater extent than the L464E substitution (Figure 5E), but the double mutation Y460E/L464E completely abrogates the IFIT1-IFIT3 interaction (Figure 5F). We also confirmed that the corresponding YxxxL motif on IFIT3 is responsible for the interaction with IFIT1 (Supplementary Figure S6). Mutation of this motif however did not affect IFIT3 homodimerization.

Disruption of dimerisation was previously reported not to affect the translation inhibition activity of IFIT1 (BioRxiv: https://doi.org/10.1101/152850 and Figure 5G, white bars). Therefore, we examined what impact mutations in the YxxxL motif had on the ability of IFIT3 to stimulate IFIT1 translation inhibition activity. IFIT1 mutants were combined with IFIT3, reporter mRNA and RRL as described in Materials and Methods and luminescence was measured (Figure 5G). At 40 nM IFIT1, YxxxL motif mutants displayed similar translation inhibition to wild type IFIT1. IFIT3 significantly enhanced translation inhibition of both wild type IFIT1 and the IFIT1-L464E mutant. In contrast, IFIT3 did not stimulate translation inhibition of either the IFIT1-Y460E mutant or the L464E/Y460E double mutant.

### IFIT2 and IFIT3 stabilize IFIT1 expression in cells

**Figure 6.**
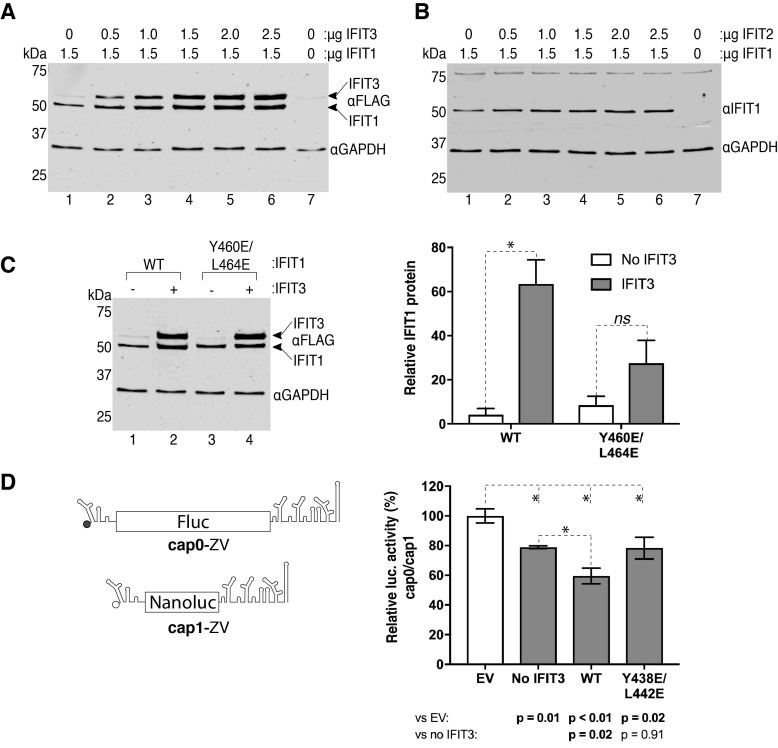
IFIT3 stabilizes and promotes IFIT1 activity in cells. (**A** and **B**) HEK293T cells were transfected with the indicated amounts of plasmid encoding FLAG-tagged versions of IFIT1, IFIT2 and IFIT3. After 24 hours cell lysates were harvested and analyzed by western blotting. (A) anti-FLAG and (B) anti-IFIT1 blots are shown. Empty vector was used to normalize the amount of DNA used in each transfection. The blots shown are representative of three separate experiments. (**C**) HEK293T cells were transfected with 1.5 μg of FLAG-tagged wild type (WT) or mutant IFIT1 and 1.5 μg of FLAG-tagged IFIT3 or empty vector as indicated. After 24 h cell lysates were analyzed as in A. The graph on right shows the quantification of the IFIT1 protein expression relative to GAPDH probed as a loading control. Mean ± the standard deviation of three biological repeats. (**D**) Reporter luciferase was measured as described in the Materials and Methods and expressed as the ratio of Fluc (cap0) over Nanoluc (cap1) signal, normalized to the empty vector control, and shown as the mean ± the standard deviation of three biological repeats. Statistical analysis was performed using an unpaired, two-tailed Students t-test. *P*-values are indicated, and * denotes statistical significance.

After confirming that reconstituted heterocomplexes are more stable than the individual IFIT proteins *in vitro* we examined the impact of IFIT complexing in a cell-based system. HEK293T cells were transfected with either FLAG-tagged IFIT1 alone or along with increasing amounts of a plasmid encoding IFIT2 or IFIT3. The amount of FLAG-tagged IFIT1 plasmid used produces a similar level of IFIT1 expression as detected after 24 h treatment with IFN-α (Supplementary Figure S7A). The presence of IFIT3 but not IFIT2 dramatically stabilized the expression of IFIT1 (Figure 6A and B). As shown in Figure 6C, the stabilization is dependent on the integrity of the YxxxL motif. Interestingly, mutation of the YxxxL motif in IFIT1 causes a small but reproducible increase in IFIT1 expression in the absence of any other IFITs (compare lanes 1 and 3 in Figure 6C). Since FLAG-tagged IFIT1 and IFIT2 could not be separated by SDS-PAGE we used anti-IFIT1 to detect protein expression. IFIT2 expression in these samples was confirmed as shown in Supplementary Figure S7B.

Finally, we examined whether IFIT3 could enhance IFIT1 activity in cells. HEK293T cells were transfected with IFIT1 alone or co-transfected with either wild type or Y438E/L442E mutant IFIT3, which cannot bind to IFIT1 (Supplementary Figure S6). After 24 h, *in vitro* transcribed and capped mRNAs comprising the Fluc or Nanoluc reporter genes shown in Figure 6D flanked by the ZV 5′ and 3′ UTRs were transfected into the IFIT-expressing cells, as described in Materials and Methods. The ZV Fluc mRNA is the same as that used in the *in vitro* translation inhibition assays in Figure 3. The ZV Nanoluc mRNA is identical to this reporter but expresses a nano luciferase gene rather than a firefly luciferase gene. As addition of cap1 to the ZV genome renders it resistant to IFIT1 inhibition (Supplementary Figure S4), translation from the cap1 ZV Nanoluc reporter serves as an internal control for RNA transfection efficiency. Translation was quantified by measuring luminescence from the Fluc or Nanoluc reporters and expressed as a ratio of Fluc(cap0)/Nanoluc(cap1) in Figure 6D. IFIT1 alone caused a 20% reduction in cap0 RNA translation compared to empty vector transfected cells. Co-expression with wild type IFIT3 resulted in a two-fold enhancement of this inhibition. In contrast, co-expression with the Y438E/L442E mutant IFIT3 that does not bind wild type IFIT1 had no effect on IFIT1-mediated translation inhibition. These in cell experiments confirm an important role for IFIT3 in promoting the full antiviral effects of IFIT1.

## DISCUSSION

The IFIT family of ISGs are among the most highly upregulated proteins during the cellular response to viral infection and, while a role for IFITs in regulating translation has long been postulated, the mechanisms by which these proteins function are only recently being revealed. Here, we have presented, to our knowledge, the first *in vitro* reconstitution of the IFIT1:IFIT2:IFIT3 complex. This has enabled us to examine the impact of hetero oligomerisation on IFIT stability, RNA recognition and translation regulation and to understand how this complex assembles.

### IFIT assembly

**Figure 7.**
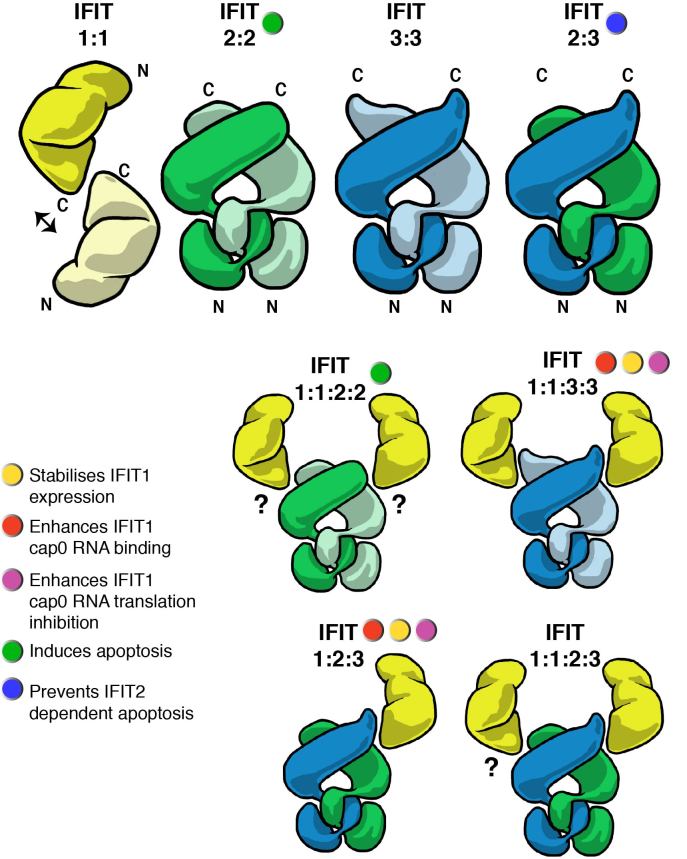
Schematic representation of the IFIT complexes analyzed in this study. Cartoons depicting IFIT1 (yellow), IFIT2 (green) and IFIT3 (blue) complexes reconstituted *in vitro* from individually purified proteins. Weak interactions are indicated by reversible arrows. IFIT1 and IFIT2 structures and dimerization interactions have been characterized by X-ray crystallography (BioRxiv: https://doi.org/10.1101/152850 and (30)). No experimentally derived structure for IFIT3 homo- or heterodimers are currently available. Therefore, IFIT3 and IFIT2:IFIT3 dimerisation, and IFIT1 heterooligomerization are modeled on the IFIT1 dimer (PDB: 5W5H) and IFIT2 dimer (PDB: 4G1T) crystal structures and supported by experimental evidence as discussed in the text. The association of IFIT1 and IFIT2 is less well defined as indicated by the question marks. A traffic light system is used to provide information on the biological roles of the different complexes.

After initially confirming that IFIT1, IFIT2 and IFIT3 interact in cell lysates in an RNA independent manner we used SEC-MALS to examine IFIT heterocomplex assembly pathways. Figure 7 shows schematic representations of each of the IFIT complexes reconstituted *in vitro* in this study.

Comparison of the different complexes assembled *in vitro* by differential scanning fluorimetry (Figure 2) reveals that although it is the least thermodynamically stable in isolation, the presence of IFIT3 enhances the stability of both IFIT1 and IFIT2. However, our results demonstrate that IFIT3 binds IFIT1 and IFIT2 in very different ways. The IFIT1 and IFIT3 interaction is rapid and occurs at low temperatures. We identified a critical YxxxL motif present in both IFIT1 and IFIT3 that is essential for interaction of the two proteins. This motif is also responsible for the concentration-dependent homodimerization of IFIT1 previously reported (BioRxiv: https://doi.org/10.1101/152850) and confirmed here. The dynamic nature of the IFIT1:IFIT1 interaction likely accounts for the ability of IFIT3 to outcompete and form the thermodynamically more stable IFIT1:IFIT3 complex. In contrast, the formation of an IFIT2:IFIT3 complex from individually purified proteins is slow and requires energy, only being detected when incubated at 37°C. This is likely due to the domain swap nature of the interaction between IFIT2 homodimers that must be disrupted for IFIT3 to instead associate. Our SEC-MALS analysis of IFIT2:IFIT3 heterodimer assembly supports this model.

While the YxxxL motif is conserved in mouse Ifit1b1, Ifit3 is truncated such that the YxxxL motif is absent (Supplementary Figure S5). In mice and other rodents, the Ifit1b1 gene has been duplicated (6) and it is possible that these extra IFITs could compensate for the disrupted murine Ifit1b1–Ifit3 interaction. This suggests species differences in the role of this motif, and in IFIT oligomerisation in general, that must be considered when examining phenotypes of small animal models used to examine the impact of IFIT depletion. Importantly, the crystal structure of IFIT5 reveals that, although critical residues of the YxxxL motif are conserved, they are buried in an interface with a terminal helix not present in IFIT1 (8–10), explaining why IFIT5 does not interact with IFIT3 ((12) and Figure 1).

Our IFIT2:IFIT3 interaction studies also have important implications for our understanding of IFIT biology. IFIT2 dimerizes through a domain swap of three α-helices that constitute one and a half TPRs of the N-terminal domain (7) (Supplementary Figure S8A and shown schematically in Figure 7). The ability of IFIT3 to sequester IFIT2 into a heterodimeric form demonstrates that IFIT3 can disrupt the domain swapped architecture of IFIT2. It is therefore possible that IFIT3 could interact with IFIT2 in a similar domain swapped manner (modeled schematically in Figure 7, *top right*), consistent with TPRs 1–4 in the N terminus of IFIT2 being sufficient for the interaction (30). Overexpression of IFIT2 induces apoptosis (30,43), while co-expression with IFIT3 but not IFIT1 blocked this effect (30). Moreover, depletion of IFIT3 induced cell death in the U549 human carcinoma cell line, an effect potentiated by co-infection with dengue virus (44). Our data would suggest that the homodimeric form of IFIT2 may be responsible for this phenotype, and that co-expression of IFIT3 can mitigate this effect by disrupting IFIT2 dimerization. It is currently not clear why the cell would evolve such a mechanism for inducing programmed cell death. One potential hypothesis is that dysregulation of ISG induction, perhaps due to infection, could perturb the balance of IFIT2 and IFIT3, promoting cell death to restrict pathogen spread. Interestingly, a poly(AU) RNA binding activity of IFIT2 was localized to the dimer interface surfaces of the C-terminal domain that form a large positively charged pocket (Supplementary Figure S8B) (7). The electrostatic surface potential of an IFIT3 molecular model, based on the IFIT2 crystal structure, is shown in Supplementary Figure S8C. The IFIT3 model lacks a positively-charged nucleic acid binding surface like that of IFIT2. Together, our assembly analysis reveals novel details about IFIT interactions and indicates a central role for IFIT3 in IFIT complex assembly.

### Impact of oligomerization on IFIT1

*In vitro* reconstitution of the human IFIT heterocomplex enabled us to examine the impact of oligomerization on the cap0 mRNA binding and translation inhibition activity of IFIT1. We opted to use an RRL based translation inhibition assay system as this was previously used to demonstrate differential inhibitory effects of IFIT1 on mRNAs from parainfluenza virus 5 (23). IFIT3 and to a lesser extent the IFIT2:IFIT3 heterodimer enhanced the translation inhibition effect of IFIT1 on a ZV reporter mRNA (Figure 3). This effect was even more pronounced when a β-globin reporter mRNA, predicted to have very little secondary structure, was analyzed consistent with an emerging consensus that both the methylation state and structure at the 5′ end of an mRNA can influence its susceptibility to IFIT1 inhibition (9,23,24). IFIT2 alone did not enhance translation inhibition by IFIT1 so it is likely that the effect observed in the presence of the IFIT2:IFIT3 complex is a result of IFIT1’s binding to IFIT3 in the complex. This can explain why the effect of the IFIT1:IFIT2:IFIT3 complex in RRL was weaker that the IFIT1:IFIT3 complex.

While multiple cellular factors such as competing cap-binding proteins complicate analysis in lysates and cells, our *in vitro* mRNA binding assays reveal that IFIT3 enhances IFIT1 cap0 mRNA binding. When examined in our reverse transcriptase inhibition assays, apparent IFIT1 binding was much less efficient for the structured cap0-ZV reporter than for the less structured β-globin mRNA and in fact on the ZV construct failed to reach saturation (Figure 4). This is not due to incomplete capping of the mRNA as saturation is possible when IFIT3 is present. Instead, it is more likely that the reverse transcriptase may remove a proportion of the bound IFIT1 as it proceeds in a 5′ to 3′ direction resulting in the production of a full-length signal even if IFIT1 was initially bound. We conclude therefore that IFIT3 stabilizes the interaction of IFIT1 with the cap0 mRNA in such a way that it is no longer removed by the reverse transcriptase consistent with its role in enhancing translation inhibition by IFIT1. Interestingly, there was no evidence of a shift in the IFIT-dependent toeprint to indicate that the other IFITs in the complex were interacting with the mRNA downstream of the IFIT1 cap0 binding cleft. IFIT5 changes conformation when transitioning between the apo- and RNA-bound state (8), positioning key residues for optimal RNA binding. The crystal structures of IFIT1 bound to different short RNAs show the protein is in a similar closed conformation as the RNA-bound structure of IFIT5 (9). It is therefore likely that IFIT1 cycles through a similar open/closed conformation to interact with RNA. Binding of IFIT3 may promote the closed conformation of IFIT1, stabilising its interaction with target RNAs.

Although the RRL system provides particular advantages for analysis of translational control by IFIT complexes it does not fully recapitulate the cellular environment that IFITs are exposed to. Using a cell-based system we observed that IFIT3 markedly stabilizes the expression of IFIT1 (Figure 6). This stabilization was dependent on the integrity of the YxxxL IFIT1:IFIT3 interaction motif. In contrast IFIT2 had a much smaller effect on IFIT1 expression in cells consistent with the stability of the different complexes observed in the *in vitro* differential scanning fluorimetry assays. Overexpression of wild type IFIT3, but not an IFIT1-binding defective mutant, also enhanced the translation inhibitory effect of IFIT1 on a model ZV reporter mRNA confirming that this interaction is functionally relevant. Therefore, through a single interaction surface IFIT3 stabilizes the expression and enhances the non-self mRNA binding activity of IFIT1.

IFIT1 puts pressure on viruses to maintain mechanisms for generating mRNAs with 5′ ends that it cannot bind. Flaviviruses for example, which have genomic RNAs ∼11000 bases in length, must dedicate a region of their limited genome to maintain a functional methyltransferase activity. The genomic 5′ UTRs of alphaviruses, which lack a methyltransferase activity, also serve as replication promoters that function more efficiently when unstructured. However, they must forfeit optimal replication to maintain a stable stem structure at the very end of their genomes to avoid IFIT1 restriction (26,27). Together, our results provide novel details about IFIT interactions and how oligomerisation affects IFIT1 stability, non-self RNA binding and translation inhibition. Our reconstituted complexes provide a solid foundation for future molecular analysis of IFIT assembly and function.

## Supplementary Material

Supplementary Data
