# Supplementary material for: IFIT3 and IFIT2/3 promote IFIT1-mediated translation inhibition by enhancing binding to non-self RNA
Source: Nucleic Acids Res. 2018 Mar 15;46(10):5269–85. doi: 10.1093/nar/gky191 (PMC6007307; doi:10.1093/nar/gky191)
Supplement: Supplementary Data [file gky191_supplemental_figures.pdf]

Supplemental Information for:

IFIT3 and IFIT2/3 promote IFIT1-mediated translation inhibition by enhancing binding to non-self RNA.

Renata C Fleith[1,2,3], Harriet V Mears[1], Xin Yun Leong[1], Thomas J Sanford[1], Edward Emmott[1#], Stephen C Graham[1], Daniel S Mansur[2], Trevor R Sweeney[1*].

1 Division of Virology, Department of Pathology, University of Cambridge, Addenbrooke's Hospital, Hills Road, Cambridge, UK.
2 Laboratory of Immunobiology, Department of Microbiology, Immunology and Parasitology, Universidade Federal de Santa Catarina, Florianópolis, Brazil.
3 Universidade Federal de Minas Gerais, Belo Horizonte, Brazil.
# Current address: Department of Bioengineering, College of Engineering, Northeastern University, 360 Huntington Ave, Boston, MA, USA.
* Address correspondence to ts629@cam.ac.uk

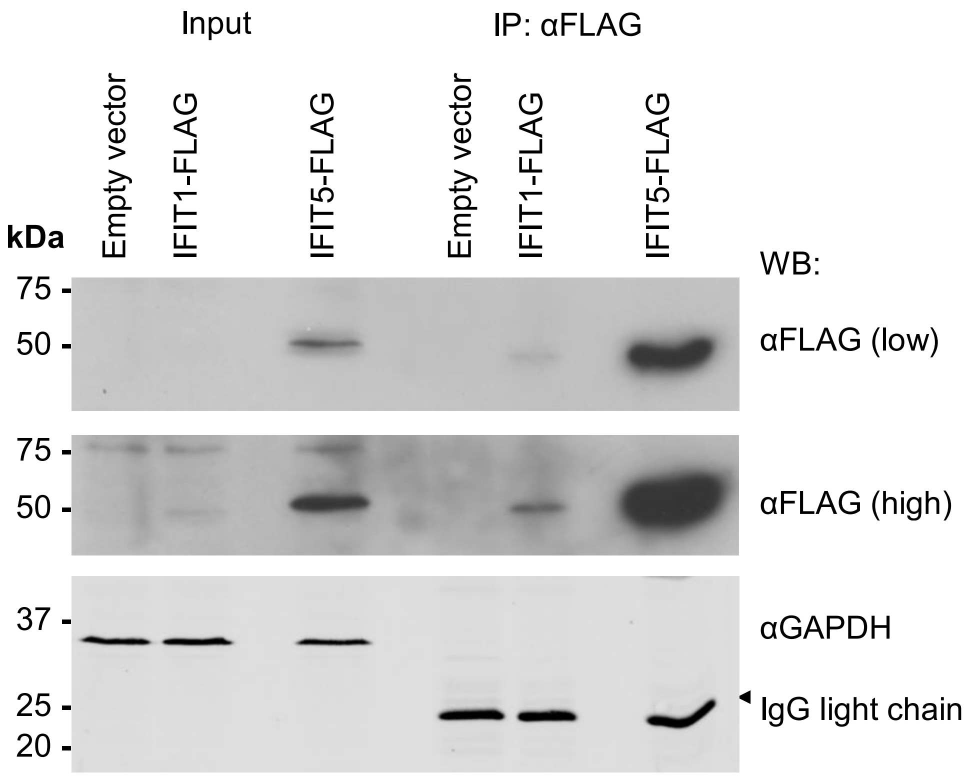

Figure S1. Anti-FLAG immunoprecipitation analysis of SILAC experiment.
HEK293T cells were passaged in differentially labelled media before being transfected with FLAG-tagged IFIT1, FLAG-tagged IFIT5 or an empty vector control for 24 hrs and subsequent treatment with IFN-α for 16 hrs. Cells were lysed and immunoprecipitated with anti-FLAG beads as described in the Materials and Methods. The input lysate and immunoprecipitated samples were analysed by western blotting with an anti-FLAG (αFLAG) or an anti-GAPDH (αGAPDH) antibody. The top and middle panels are different exposures of the same western blot that facilitate visualisation of IFIT1 in the input sample. The anti-mouse secondary antibody detects the IgG from the antibody used for the immunoprecipitation.

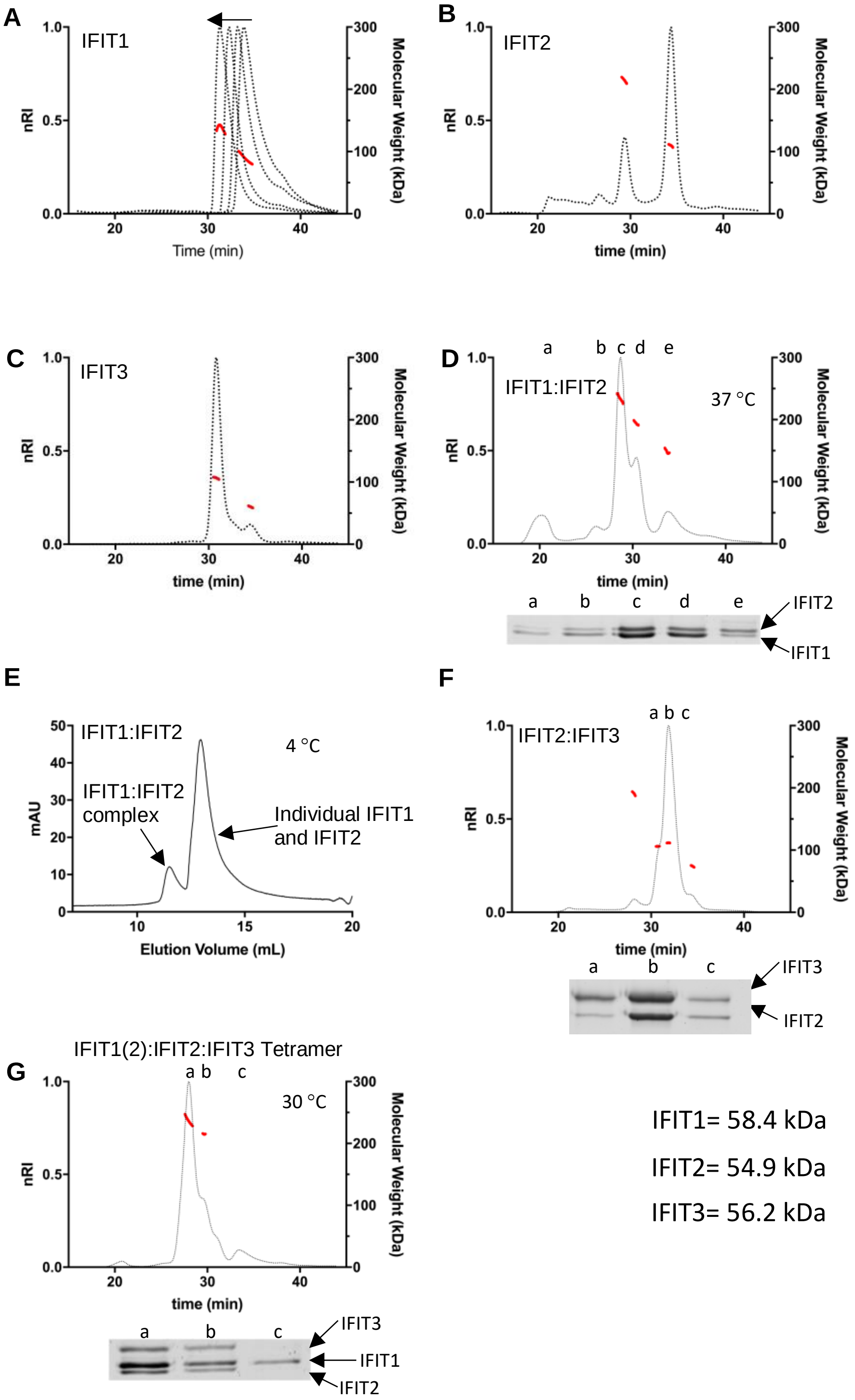

IFIT1= 58.4 kDa

IFIT2= 54.9 kDa

IFIT3= 56.2 kDa

Figure S2. SEC and SEC-MALS analysis of IFIT1, IFIT2, IFIT3 and IFIT1 complexes.
(**A-D, F, G**) Purified individual IFITs or their complexes were loaded onto a Superdex200 10/300 Increase size exclusion column and the mass of eluting species monitored by MALS as described in the Materials and Methods. (**A**) IFIT1 (0.5, 1, 2.8 or 8 mg/ml, arrow indicates earlier elution of peak with increasing amount of IFIT1 loaded onto column), (**B**) IFIT2 (1 mg/ml), (**C**) IFIT3 (1 mg/ml) and (**D**) 1 mg/ml each of IFIT1 and IFIT2 preincubated for 1 hour at 30 °C were analysed by SEC-MALS. (**F**) The IFIT2:IFIT3 peak fractions shown in Figure 2C were reanalysed by SEC-MALS. (**G**) The IFIT2:IFIT3 complex shown in F was incubated with a two-fold molar excess of IFIT1 and analysed by SEC-MALS. (A-D, F, G) dotted line represents the normalised differential refractive index (nRI) of the sample eluting from the size exclusion column. Red solid line shows the calculated molecular mass of the species eluting in each peak. The gel inset shows the SDS-PAGE analysis of the proteins eluting in each peak. While the most abundant IFIT1:IFIT2 complex form is likely tetrameric, the presence of several overlapping peaks precludes reliable determination of specific molecular mass and hence oligomeric state.The calculated molecular masses of individual IFITs are: IFIT1(with 6His tag)- 58.4 kDa, IFIT2- 54.9 kD and IFIT3- 56.2 kDa. (**E**) 1 mg/ml each of IFIT1 and IFIT2 preincubated for 1 hour at 4 °C were analysed by SEC.

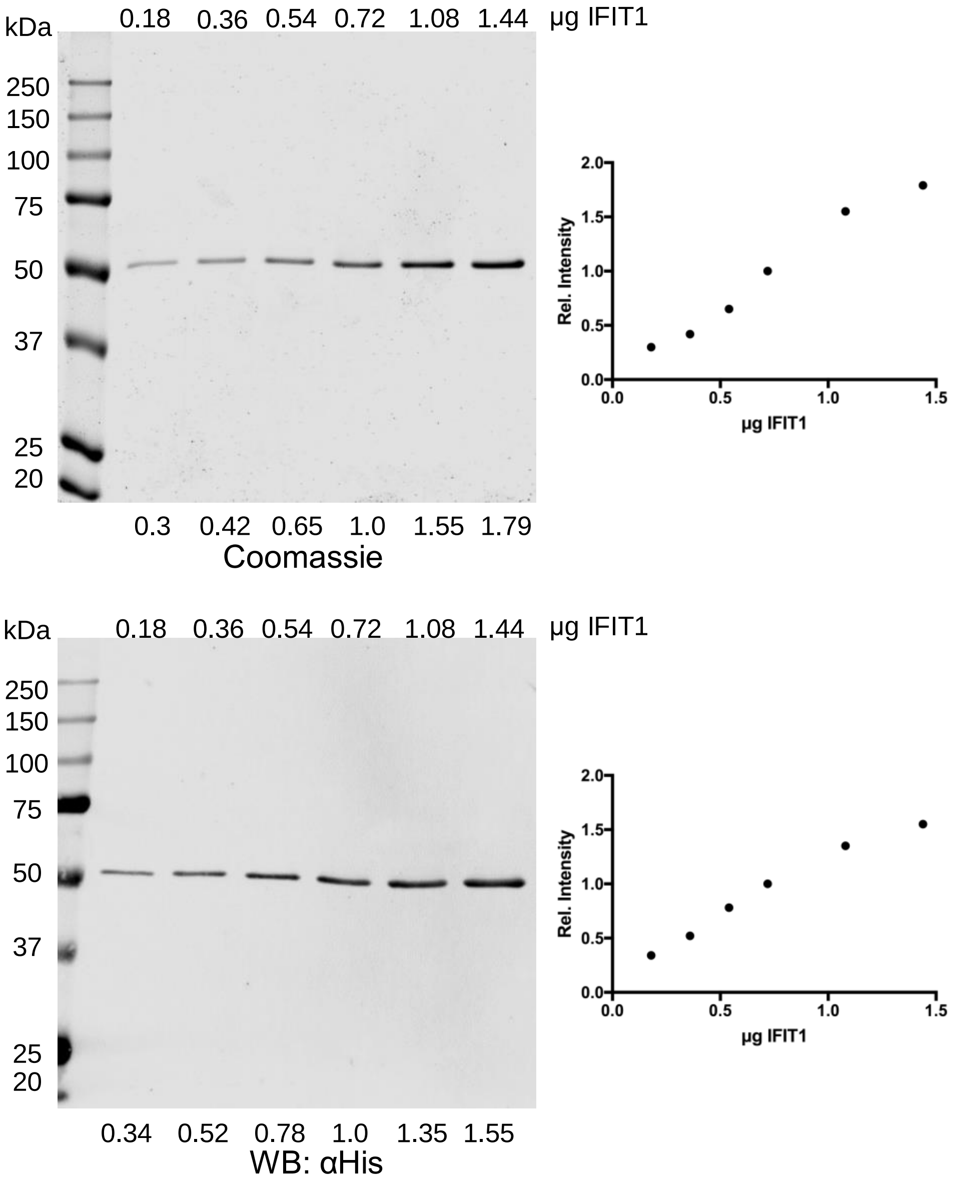

Figure S3. Analysis of IFIT1 by western blot.

The indicated amounts of purified IFIT1 were separated by SDS-PAGE and visualised by Coomassie staining or by western blot (WB) analysis using an anti-His-tag antibody as described in the Materials and Methods. The relative (Rel.) intensity of the Coomassie stained gel and anti-His signal was measured in ImageJ and indicated at the bottom of the gel and graphed on the right. Concentration of IFIT1 alone or in the complexes was normalised based on measurements where changes in protein concentration and signal intensity were linear.

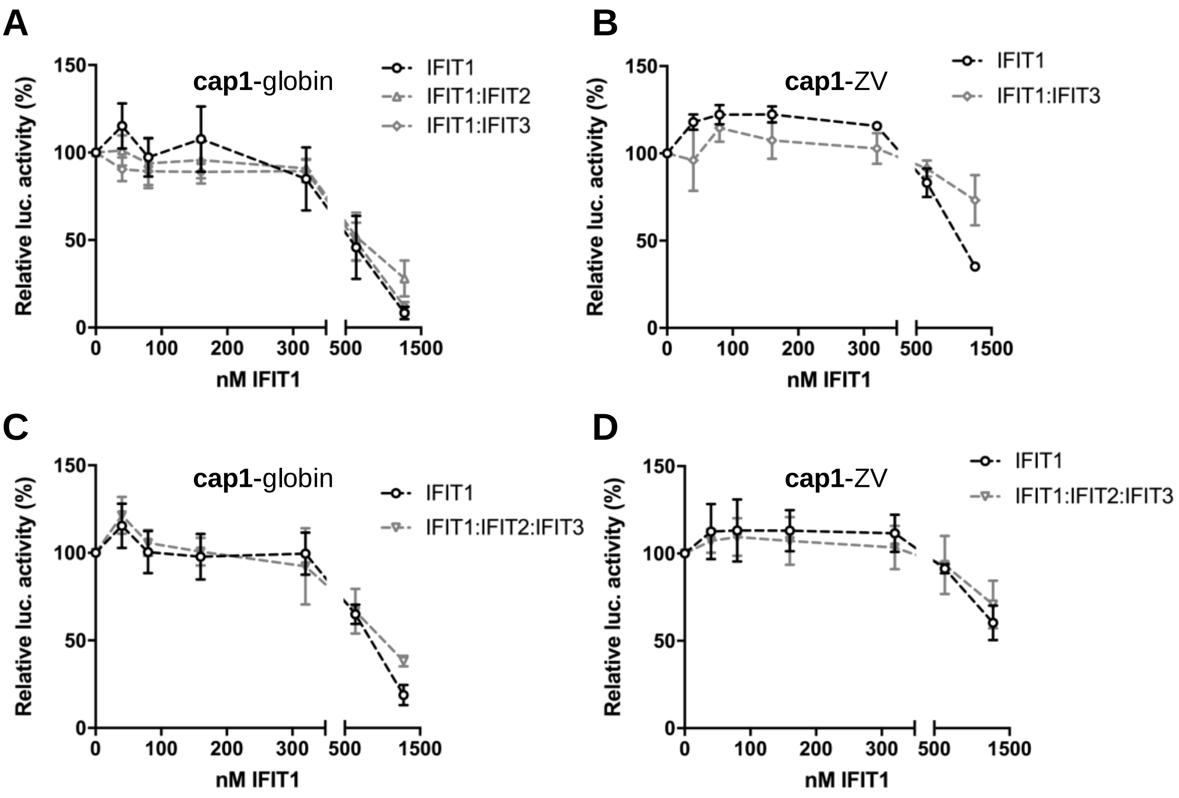

Figure S4. Inhibition of cap1 dependent translation by IFIT complexes in RRL.
The indicated IFIT1 containing complexes were incubated with (**A**, **C**) cap1-globin Fluc reporter mRNA or (**B**, **D**) cap1-ZV Fluc reporter mRNA in RRL. Translation was monitored by luciferase activity. Data are presented as percentage luciferase activity relative to the no IFIT1 control ± SEM, based on three separate experiments.

```
IFIT1       MSTNGDDHQVKDSLEQLRCHFTWELSIDDDEMPDLENRVLDQIEFLDTKYSVGIHNLLAY  60
Ifit1b1     MGENADGDQVMENLLQLRCHFTWKLLFENNDIPDLEVRISEQVQFLDIKNPLGMHNLLAY  60
IFIT3       MSE-VTKNSLEKILPQLKCHFTWNLFKEDSVSRDLEDRVCNQIEFLNTEFKATMYNLLAY  59
Ifit3       MSE-VNRESLEAILPQLKCHFTWNLFREGSMSSHMEDRVCNQVEHLNSEEKATMYDLLAY  59
            *.     ..:   * **:*****:*  :..   .:* *: :*::.*: :    :::****

IFIT1       VKHLKGQNEEALKSLKEAENLMQEEHDNQANVRSLVTWGNFAWMYYHMGRLAEAQTYLDK  120
Ifit1b1     VRHLKGQQDEALQSLKEAEALIQSE---QLSKRSLATWGNCAWLHYHRGSLAEAQIYLDK  117
IFIT3       IKHLDGNNEAALECLRQAEELIQQEHADQAEIRSLVTWGNYAWVYYHLGRLSDAQIYVDK  119
Ifit3       IKHLDGESKAALECLGQAEDLRKSEHNDQSEIRRLVTWGNYAWIYYHMGRLSEAQAYVDK  119
            ::**.*:.. **:.* :** * :.*   * . * *.**** **::** * *::** *:**

IFIT1       VENICKKLSNPFRYRMECPEIDCEEGWALLKCGGKNYERAKACFEKVLEVDPENPESSAG  180
Ifit1b1     VEKVCKEFSSPFRYRLECAEMDCEEGWALLKCGGGNYKQAMACFAKALKVEPENPEYNTG  177
IFIT3       VKQTCKKFSNPY--SIEYSELDCEEGWTQLKCGRN--ERAKVCFEKALEEKPNNPEFSSG  175
Ifit3       VRQVCQKFANPY--SMECPELECEEGWTRLKCGRN--ERAKMYFEKALEEKPKDPECSSG  175
            *.: *::::.*:   :*  *::*****: ****    ::*   * *.*: .*::** .:*

IFIT1       YAISAYRLDGFKLATKNHKPFSLLPLRQAVRLNPDNGYIKVLLALKLQDEGQEAEGEKYI  240
Ifit1b1     YAVVAYRQDL------DDNFISLEPLRKAVRLNPEDPYLKVLLALKLQDLGEHVEAEAHI  231
IFIT3       LAIAMYHLDN-----HPEKQFSTDVLKQAIELSPDNQYVKVLLGLKLQKMNKEAEGEQFV  230
Ifit3       MAIAMFRLEE-----KPEKQFSVDALKQAMELNPQNQYLKVLLALKLLRMGEEAEGERLI  230
             *:  :: :        .: :*   *::*:.*.*:: *:****.***   .:..*.*  :

IFIT1       EEALANMSSQTYVFRYAAKFYRRKGSVDKALELLKKALQETPTSVLLHHQIGLCYKAQMI  300
Ifit1b1     EEALSSTSCQSYVIRYAAKYFRRKHRVDKALHLLNRALQASPSSGYLHYQKGLCYKQQIS  291
IFIT3       EEALEKSPCQTDVLRSAAKFYRRKGDLDKAIELFQRVLESTPNNGYLYHQIGCCYKAKVR  290
Ifit3       KDALGKAPNQTDVLQKAAQFYKKKGNLDRAIELLGKALRSTVNNSPLYSLVMCRYREILE  290
            ::** .   *: *:: **:::::*  :*:*:.*: :.*. : ..  *:      *:  :

IFIT1       QIKEATKGQPRGQNREKLDKMIRSAIFHFESAVEKKPTFEVAHLDLARMYIEAGNHRKAE  360
Ifit1b1     QLRTSRNRQPRRQD--NVQELAQQAIHEFQETLKLRPTFEMAYVCMAEVQAEIHQYEEAE  349
IFIT3       QMQNTGESEASG-NKEMIEALKQYAMDYSNKALEKGLNPLNAYSDL-------AEFLET-  341
Ifit3       QLQNKGDADSSE-RRQRMAELRRLTMEFMQKTLQRRRSPLNSYSDL-------IDFPEVE  342
            *::   . :        :  : : ::   :.:::   .   ::  :        :. :.

IFIT1       ENFQKLLCMKPVVEETMQDIHFHYGRFQEFQKKSDVNAIIHYLKAIKIEQASLTRDKSIN  420
Ifit1b1     RNFQKALNNKTLVAHIEQDIHLRYGRFLQFHKQSEDKAITLYLKGLKVEEKSFAWRKLLT  409
IFIT3       ECYQTPF-NKEVPDAEKQQSHQRYCNLQKYNGKSEDTAVQHGLEGLSISKKSTDKEEIKD  400
Ifit3       RCYQMVI-SKESPDVEEEDLYERYCNLQEYHRKSEDLAALECLLQFPRNERSIEKEEVKE  401
            . :*  :  *       ::  : :* .: ::: :*:  *    *  :  .:  *    :

IFIT1       SLKKLVLRKLRRKALDLESLSLLGFVYKLEGNMNEALEYYERALRLAADFENSV------  474
Ifit1b1     ALEKVAERRVCQNVHLVESTSLLGLVYKLKGQEKNALFYYEKALRLTGEMNPAF------  463
IFIT3       QPQNVSENLLPQNAP--NYWYLQGLIHKQNGDLLQAAKCYEKELGRLLRDAPSGIGSIFL  458
Ifit3       QT----------------------------------------------------------  403

IFIT1       ------------RQGP----------------    478
Ifit1b1     --------------------------------    463
IFIT3       SASELEDGSEEMGQGAVSSSPRELLSNSEQLN    490
Ifit3       --------------------------------    403
```

Figure S5. Alignment of human and murine IFIT1 and IFIT3.

The sequences of IFIT1 and IFIT3, and mouse Ifit1b1 and mouse Ifit3 were aligned using Clustal Omega. The IFIT1:IFIT3 dimerisation motif is boxed while residues conserved between IFIT1, IFIT3 and Ifit1b1 are in green.

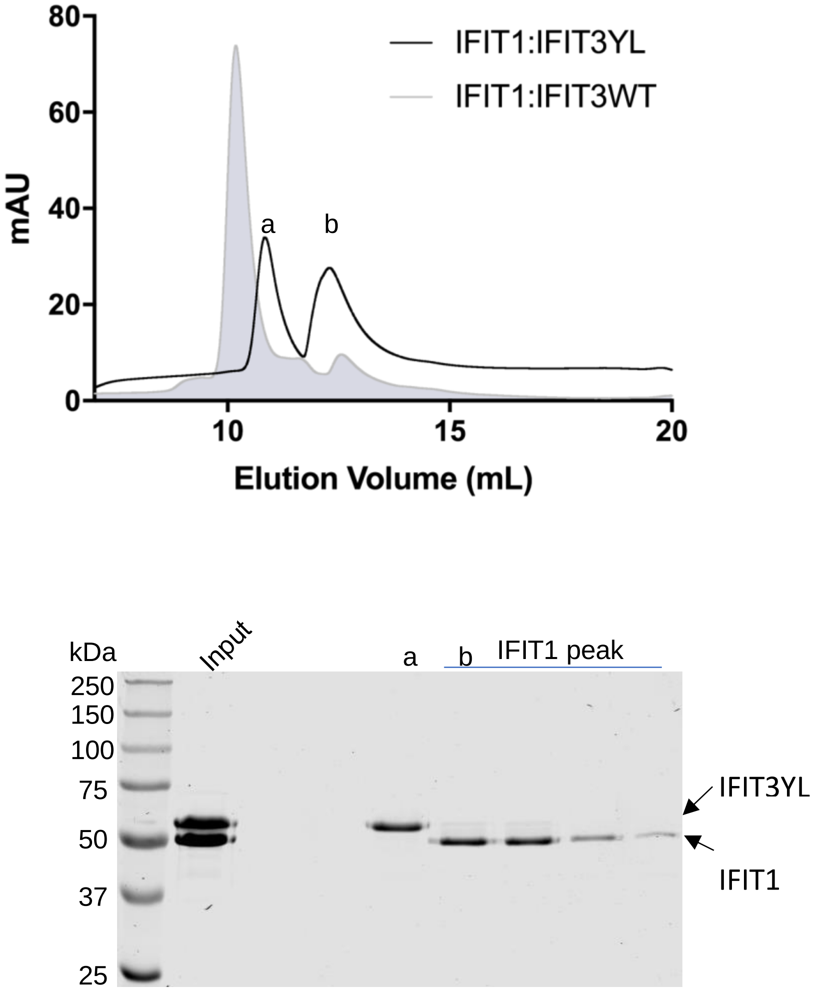

Figure S6. Mutation of IFIT3 YxxxL motif disrupts interaction with IFIT1.
Purified wt IFIT1 and IFIT3-Y438E/L442E (IFIT3YL) were preincubated for 1 hour at 4 °C and loaded onto a Superdex200 10/300 Increase size exclusion column. The absorbance at 280 nm is shown in the upper panel. The grey line shows the trace obtained when wt IFIT:IFIT3 are analysed using the same method. The IFIT3 YL mutation disrupts interaction with IFIT1 and so the proteins elute as two separate peaks. Analysis of the peak fractions by SDS-PAGE is shown in the bottom panel. a and b correspond to the proteins present in the peaks indicated in the upper panel. No IFIT1 is found in the IFIT3YL peak. The IFIT3YL still migrates as a dimer.

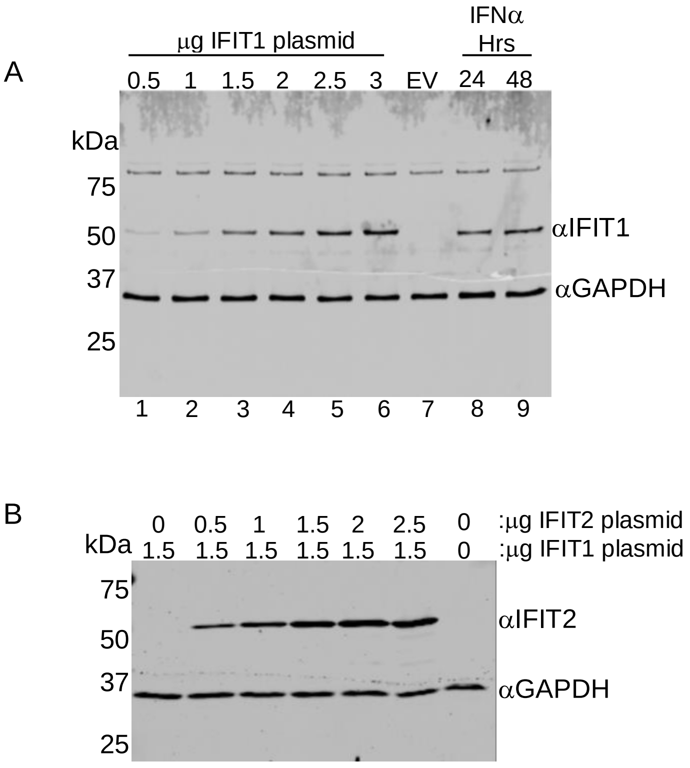

Figure S7. Overexpression of IFIT1 and IFIT2 in HEK293T cells

(**A**) *Lanes 1-7*, HEK293T cells were transfected with empty vector (EV) or increasing concentrations of FLAG tagged IFIT1 for 24 hrs before lysates were collected and analysed by western blotting using an anti-IFIT1 antibody. Empty vector was added to normalise the amount of DNA transfected. *Lanes 8-9*, HEK293T cells were separately treated with 1000 U/mL human IFN-α for 24 or 48 hrs and cell lysates were collected and analysed alongside the overexpressed samples. (**B**) This panel relates to Figure 6B in the main text. HEK293T cells were transfected with the indicated amounts of plasmid encoding FLAG-tagged versions of IFIT1 and/or IFIT2. Empty vector was added to normailise the amount of DNA transfected. After 24 hours cell lysates were harvested and analysed by western blotting. As FLAG-tagged IFIT1 and IFIT2 could not be separated by SDS-PAGE we probed the same samples with anti-IFIT2 antibody, here, and anti-IFIT1 antibody (Figure 6B, main text). Blots are representative of three separate experiments.

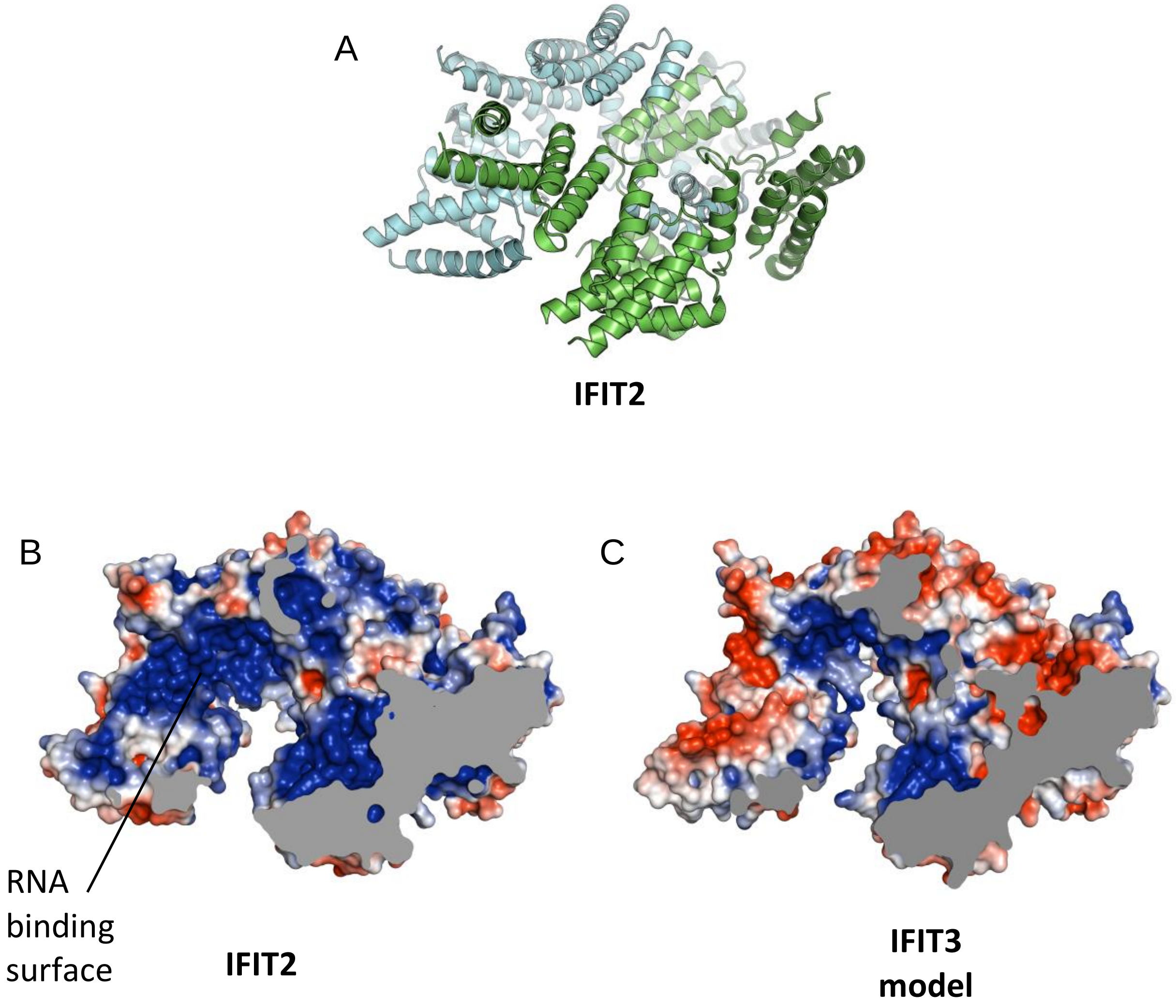

Figure S8. Modelling of the differing electrostatic surface potential of IFIT2 and IFIT3. (**A**) Crystal structure of IFIT2 dimer (PDB: 4G1T) showing the individual chains a, in green and b, in cyan. (**B**) Electrostatic potential (blue= positive, red= negative) of IFIT2 calculated using APBS. The viewpoint is the same as in A, but chain a is partially removed to expose the highly positively charged surface at the interface between the two chains. (**C**) A model of IFIT3 was generated by the Swiss Model server using IFIT2 (PDB:4G1T) as a template. The model is shown in the same orientation as for IFIT2 in A and one chain has been removed in a similar manner to B to reveal the very different surface potential of IFIT3 at the modelled dimer interface. Structure analysis and images were generated using PyMOL (The PyMOL Molecular Graphics System, Version 2.0 Schrödinger, LLC).

Full gels from which insets in the main figures were cropped.

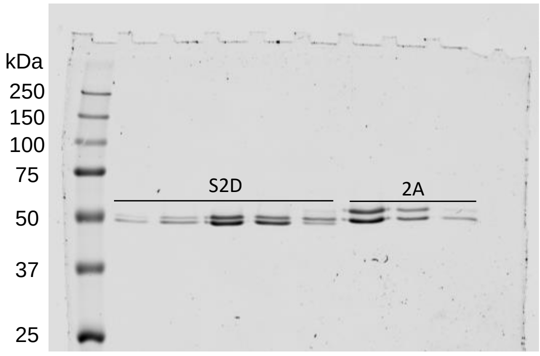

Gel used for Figure S2D and Figure 2A

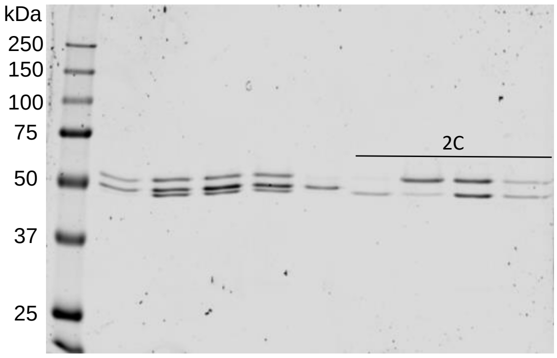

Gel used for Figure 2C

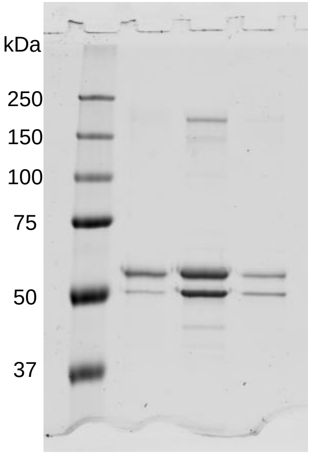

Gel used for Figure S2F

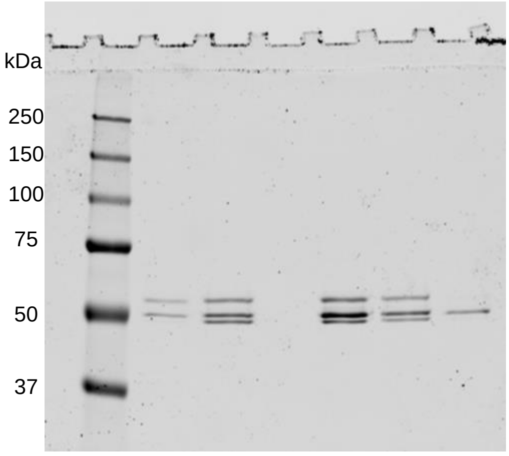

Gel used for Figures 2E and S2G

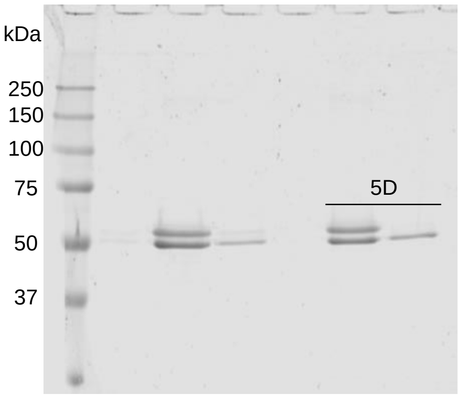

Gel used for Figure 5D

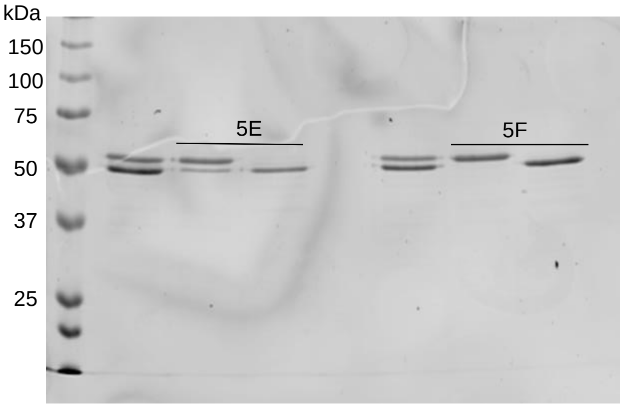

Gel used for Figure 5E and F
